# Label-free biochemical imaging and time point analysis of neural organoids via deep learning–enhanced Raman microspectroscopy

**DOI:** 10.1126/sciadv.aec5080

**Published:** 2026-08-12

**Authors:** Dimitar Georgiev, Ruoxiao Xie, Daniel Reumann, Xiaoyu Zhao, Álvaro Fernández-Galiana, Mauricio Barahona, Molly M. Stevens

**Affiliations:** ^1^ Department of Computing, and UKRI Centre for Doctoral Training in AI for Healthcare, Imperial College London, London, UK SW7 2AZ.; ^2^ Department of Materials, Department of Bioengineering, and Institute of Biomedical Engineering, Imperial College London, London, UK SW7 2AZ.; ^3^ Department of Physiology, Anatomy and Genetics, Department of Engineering Science, and Kavli Institute for Nanoscience Discovery, University of Oxford, Oxford, UK OX1 3QU.; ^4^ Department of Engineering Science, University of Oxford, Oxford, UK OX1 3PJ.; ^5^ Department of Mathematics, Imperial College London, London, UK SW7 2AZ.

## Abstract

Three-dimensional (3D) organoids have emerged as powerful models for studying human development, disease, and drug response in vitro. Yet, their analysis remains constrained by standard imaging and characterization techniques, which are invasive, require exogenous labeling, and offer limited multiplexing. Here, we present a noninvasive, label-free imaging platform that integrates Raman microspectroscopy with deep learning–based hyperspectral unmixing for unsupervised, spatially resolved biochemical analysis of neural organoids. Our approach enables 2D and 3D mapping of cellular and subcellular structures in both cryosectioned and intact organoids, achieving improved imaging accuracy and robustness compared to conventional methods for hyperspectral analysis. Using our platform, we demonstrate volumetric imaging of a neural rosette within a neural organoid and interrogate changes in biochemical composition during early developmental stages in intact neural organoids, revealing spatiotemporal variations in lipids, proteins, and nucleic acids. This work establishes a versatile framework for high-content, label-free (bio)chemical phenotyping with broad applications in organoid research and beyond.

## INTRODUCTION

The ability to image and accurately characterize the architecture and molecular composition of cells and tissues has been central to advancing biological and medical sciences. To this end, a broad array of techniques—ranging from histological staining and immunofluorescence to omics-based profiling—has been developed, each offering distinct insights across spatial and molecular scales (*1*–*6*). Nonetheless, a fundamental challenge persists: Most available methods are inherently invasive and/or require molecular labeling.

These constraints become particularly limiting as scientific research shifts toward complex, three-dimensional (3D) tissue models, such as organoids. Over the past decade, neural organoids derived from pluripotent stem cells have emerged as powerful biological models of the developing human brain, recapitulating key features of early developmental dynamics and organization (*7*–*10*). Compared to 2D neural cell culture models, neural organoids exhibit enhanced complexity and 3D structures, providing a more advanced representation of tissue architecture (*7*, *10*), cellular diversity (*11*, *12*), and functional network formation (*13*). Consequently, neural organoids have spurred new opportunities for studying human brain development, disease, and drug response in vitro (*14*–*17*). Yet, despite growing interest, organoid research remains constrained by the limitations of conventional methods for tissue imaging and characterization. There is a pressing need for techniques that can interrogate spatial organization and dynamics in intact organoid systems under physiologically relevant settings without prior knowledge of specific molecular targets or the introduction of exogenous labels.

Currently, immunofluorescence staining combined with fluorescence microscopy is widely used as a gold standard, reference technique for visualizing the morphological organization of 2D and 3D cellular models, owing to its high specificity and spatial resolution (*18*, *19*). In this approach, fluorophore-conjugated antibodies are used to selectively bind to and visualize specific target molecules (*20*). Despite its strengths, however, this technique has several inherent limitations. First, while protein labeling is generally well established, detecting other biomolecular classes, such as carbohydrates, lipids, and various small molecules (e.g., drugs and metabolites), often requires more specialized probes or labeling strategies or is not possible altogether. In addition, immunofluorescence staining involves multiple washing and permeabilization steps, which can disrupt cellular architecture and compromise membrane integrity, potentially leading to artefactual results (*21*). These steps may also displace small molecules such as drugs, metabolites, and other transient compounds, obscuring their true spatial localization in situ within cells and tissues. While other labeling strategies, such as fluorescent protein tagging, genetic modifications, and dye-based staining, may help avoid these washing steps, they remain label based (i.e., targets must be known and available), and issues with labeling nonproteins persist. Furthermore, regardless of the labeling strategy, the intrinsic spectral overlap of fluorescent markers constrains multiplexing, typically to four targets in standard fluorescence microscopy (*22*), or seven in more advanced techniques (*23*, *24*). Moreover, genetic modifications used in live-cell imaging to express fluorescent protein reporters can be costly, time-consuming, and may induce poorly understood side effects, such as protein aggregation or disruption of ion transport dynamics (*25*, *26*). Enabling noninvasive, label-free analysis would thus be essential to expand the potential of neural organoids in neurodevelopmental research, disease modeling, drug screening, and beyond.

Raman spectroscopy (RS), a technique within vibrational spectroscopy, offers a promising alternative for biochemical analysis. Unlike fluorescence-based imaging, RS provides molecular contrast through the analysis of the intrinsic inelastic light scattering of molecules, thereby enabling noninvasive, label-free chemical characterization with minimal sample preparation (*27*–*29*). Consequently, RS has become a ubiquitous analytical technique across the life and physical sciences (*30*–*40*), with increasing use in analyzing biological specimens such as cells and tissues (*41*–*46*). In the context of neurodevelopment, RS has been applied to profile neural cells at various stages of differentiation. Yet, most studies to date have focused on 2D cultures of individual cell types with RS measurements acquired at a single-cell level—e.g., distinguishing neural stem cells from neurons by identifying distinct cell-specific spectral signatures (*47*–*51*). The application of RS to complex 3D cultured tissues containing multiple cell types, such as neural organoids, remains challenging because of their high biochemical heterogeneity, which hinders signal unmixing and interpretation. Recently, Bruno *et al.* (*52*) explored the use of supervised machine learning to classify RS spectra from cortical organoids at four predefined maturation time points (weeks 6, 12, 16, and 20). However, their approach focused on single-point prediction and did not explore the 3D architecture, cellular interactions, and functional complexity intrinsic to neurodevelopment. So far, the use of RS for spatially resolved analysis of neural organoid structure and maturation remains unexplored.

Our laboratory recently reported a technique based on RS for 3D biochemical imaging and drug distribution analysis in liver organoids (*53*). The proposed methodology relied on the acquisition of “tissue phantoms”—i.e., calibrated reference spectra from biomolecules of interest, which were used to guide the estimation of chemical concentration maps across the scanned area using ordinary least squares with nonnegativity constraint (*54*). This enabled the detection and visualization of key biomolecules and drugs in situ in whole liver organoids. Nonetheless, the approach has two limitations. First, it assumes that target biomolecules are known, thereby restricting potential applications and undermining the label-free capabilities of RS. Second, the performance and robustness of ordinary least squares for chemical estimation can be compromised by complex mixtures, experimental noise and artefacts, and variations introduced during data calibration and preprocessing, leading to inaccurate qualitative and quantitative analyses. To address these limitations, we recently introduced a deep learning framework for RS chemometrics based on hyperspectral unmixing autoencoder neural networks (*55*). The approach enabled fully unsupervised, label-free (bio)chemical analysis with enhanced accuracy compared to standard chemometric techniques, as validated on synthetic mixtures and experimental data from sugar mixtures and a THP-1 cell.

Building on this work, we present here a pipeline for noninvasive, label-free biochemical imaging of both cryosectioned and intact neural organoids based on spontaneous Raman microspectroscopy and unsupervised autoencoder-based hyperspectral unmixing analysis. We show that our approach achieves improved organoid imaging compared to standard methods for RS analysis, as validated against fluorescence microscopy, and enables detailed morphochemical imaging and spatially resolved analysis in 2D and 3D with subcellular resolution. Using our platform, we demonstrate volumetric in situ imaging of a neural rosette structure within a neural organoid—a key morphological feature of the developing nervous system—and interrogate changes in biochemical composition during early developmental stages in intact neural organoids, revealing spatiotemporal variations in lipids, proteins, and nucleic acids.

## RESULTS

### Deep learning–enhanced Raman microspectroscopy

We begin by discussing the technical methodology behind our organoid imaging pipeline based on Raman microspectroscopy combined with autoencoder-based hyperspectral unmixing. Figure 1A provides an overview of our approach.

**Fig. 1.**
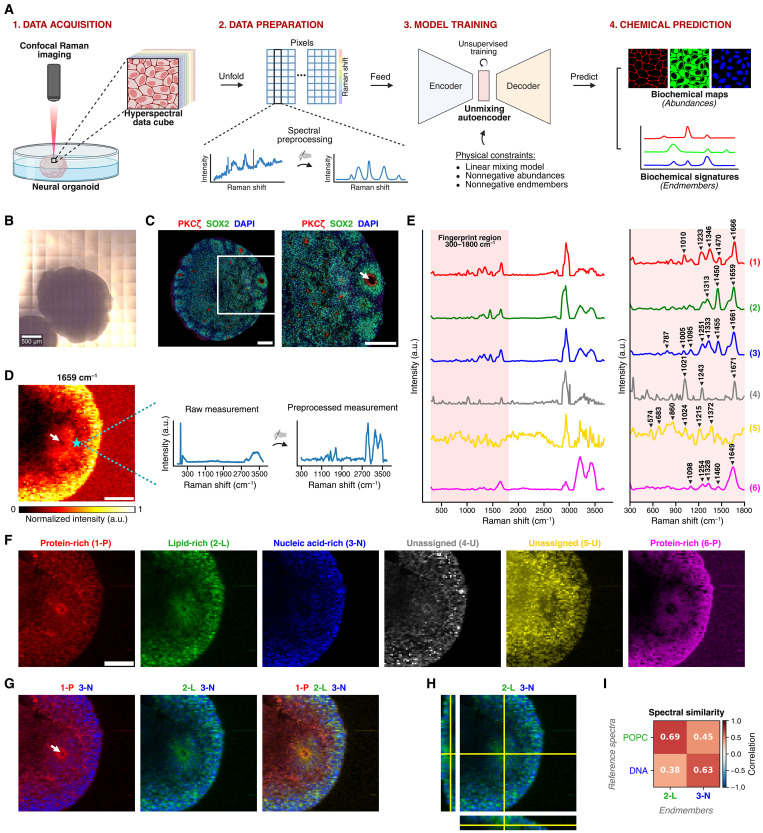
Deep learning–enhanced Raman microspectroscopy enables label-free in situ biochemical imaging of intact neural organoids in 2D and 3D. (**A**) Schematic diagram of our organoid imaging pipeline combining Raman microspectroscopy and deep learning–enhanced hyperspectral analysis. (**B**) Bright-field image of a representative day-20 neural organoid sample. Scale bar, 500 μm. (**C**) Fluorescence microscopy image of a representative day-20 neural organoid sample—full organoid sample (left), and a zoomed-in region (right), with staining markers for apical domains (PKCζ), neural progenitor cells (SOX2), and nuclei (DAPI). Arrow indicates the lumen of a present neural rosette. Scale bar, 200 μm. (**D**) Snapshot of collected volumetric Raman imaging data from an intact day-20 neural organoid, showing the intensity profile at the 1659 cm^−1^ band (proteins and lipids) of a selected *z* layer (left), and a representative tissue spectrum before and after preprocessing (right). Arrow indicates the lumen of a present neural rosette. Scale bar, 100 μm. a.u., arbitrary units. (**E** to **H**) Results of hyperspectral unmixing analysis. (E) Derived tissue-specific endmember signatures—whole wavenumber range (left) and fingerprint region only (right). Endmembers scaled for visualization purposes (maximum intensity set to 1). [(F) and (G)] Corresponding abundance maps for the same *z* layer as in (D), including single-channel images (F) and representative multichannel images (G). Arrow indicates the lumen of the present neural rosette [also shown in (D)]. Scale bar, 100 μm. (H) Volumetric abundance reconstruction of two selected endmembers linked to lipid- and nucleic acid–rich species. (**I**) Heatmap showing the Pearson correlation coefficients between derived lipid- and nucleic acid–rich endmembers and reference spectra of POPC (phospholipid) and DNA. Reference spectra from LaLone *et al.* (*53*). Raman data were acquired using the following parameters: pixel size = 5 μm in *x*-*y* and 10 μm in *z*, integration time = 0.5 s, laser power ≈ 32 mW, objective = 63×, numerical aperture (NA) 1. Created in BioRender. Georgiev, D. (2026) https://BioRender.com/n77j3vq.

Raman measurements were collected from fixed neural organoid samples by performing point-wise imaging scans across predefined regions of interest (ROIs). Volumetric scans were generated by acquiring sequential scans along the axis perpendicular to the imaging plane (e.g., sampling at different depths along the *z* axis). Acquired data were preprocessed to remove nonspecific signal contributions and spectral artefacts, such as cosmic spikes, autofluorescence baselines, and dark noise (see Materials and Methods). This was done using RamanSPy—an open-source Python toolbox for RS data analysis we recently developed (*56*).

After preprocessing, hyperspectral unmixing was performed to derive constituent spectral components (endmembers) and quantify their relative contributions (abundances) in each measurement (*57*, *58*). This was achieved using a custom autoencoder neural network model designed for hyperspectral unmixing. The model builds upon our previously reported approach (*55*), with extensions and optimizations across the entire pipeline, spanning preprocessing, network architecture, training strategy, and endmember initialization (see Materials and Methods for more details). Briefly, the autoencoder architecture included (i) an encoder responsible for abundance estimation, which comprised a multibranch spectral convolutional block followed by a five-layer fully connected network, and (ii) a one-layer decoder responsible for endmember identification. Our model incorporated relevant physical constraints in the representation learning process, namely, constraining the learned endmembers and abundances to be nonnegative through the use of appropriate activation functions and weight clipping, and constraining the mixing process to linear mixing through the use of a single-layer linear decoder. Models were trained on individual scans or datasets in an unsupervised manner by minimizing a reconstruction loss based on the weighted sum of the mean squared error and spectral angle divergence (*59*) between input spectra and output reconstructions. The number of endmembers to extract for each scan/dataset was estimated using principal component analysis (PCA) (see Methods and Materials) (*60*, *61*).

After model training, learned endmember signatures and their corresponding abundances were derived, generating multichannel abundance images/volumes with channels rendering chemical maps of the distribution of learned endmembers. The obtained endmembers and abundances were examined to filter out components linked to nontissue signals, such as background and noise. The remaining tissue-specific endmembers were characterized via peak assignment, whereby prominent peaks were linked to chemical bonds to identify contributions from essential biomolecular species, such as nucleic acids, proteins, lipids, and carbohydrates. Peak assignment was guided by established reference materials (*42*). The corresponding abundance maps were visualized as morphochemical images showing the spatial distribution of the obtained chemical components to study organoids’ morphology and biochemical composition. Further technical details regarding data acquisition, preprocessing, and unmixing analysis are presented in Materials and Methods.

### Label-free imaging of intact neural organoids via deep learning–enhanced Raman microspectroscopy

We investigated the capabilities of our platform using Raman imaging data from neural organoids cultured in-house following an unguided differentiation protocol (fig. S1 and Materials and Methods) (*7*). Bright-field microscopy revealed spherical neural organoid samples reaching about 1 to 2 mm in size after 20 days in culture (Fig. 1B). Furthermore, fluorescence microscopy showed characteristic morphology with neural rosettes readily visible on day 20 (Fig. 1C).

As a demonstration of our Raman imaging platform, we performed volumetric Raman imaging of a tissue region within an intact day-20 neural organoid. Specifically, a *z*-stack was acquired with an axial step size of 10 μm in *z* (organoid depth), with each layer representing an 80-by-80 imaging scan with a lateral pixel size of 5 μm in the *x*-*y* plane. Five consecutive layers along the *z* axis were used for unmixing analysis, covering tissue areas up to approximately 70 μm deep into the sample. Figure 1D shows the intensity profile at 1659 cm^−1^ of one of the five layers after preprocessing, a band associated with proteins (amide I) and lipids (C=C stretching). Although the overall outline of the organoid was visible, finer structures were difficult to resolve using univariate analysis of single Raman bands.

To address this issue, we performed multivariate hyperspectral unmixing using our deep learning-enhanced Raman microspectroscopy pipeline. Our analysis yielded six tissue-specific spectral endmembers, which we characterized via peak assignment into major biomolecular classes based on characteristic Raman vibrations in the fingerprint region from 300 to 1800 cm^−1^ (Fig. 1E and extended data in fig. S2). The corresponding abundance maps were visualized as morphochemical images of the scanned organoid sample (Fig. 1, F and G).

Endmember 1 (red) exhibited a spectral signature characteristic of protein species, with prominent peaks at 1010 cm^−1^ (phenylalanine), 1233 cm^−1^ (amide III), 1346 cm^−1^ (CH deformation), 1470 cm^−1^ (CH_2_ bending, C=N stretching), and 1666 cm^−1^ (amide I). The abundance map showed broad distribution throughout the organoid tissue, likely corresponding to cytoplasmic regions. Slightly increased intensity was observed around a structure resembling the apical domain of a neural rosette, possibly indicating membrane-associated protein structures.

Endmember 2 (green) was characterized by strong lipid-associated peaks at 1313 cm^−1^ (CH_2_/CH_3_ twisting), 1450 cm^−1^ (CH_2_ bending), and 1659 cm^−1^ (C=C stretching), suggesting the presence of lipid-rich compartments such as cellular membranes and lipid bodies. This component was similarly broadly distributed across the tissue, with increased signal in the outer layers of the sample, and around the apical domain of the neural rosette, forming a radially oriented pattern.

Endmember 3 (blue) displayed canonical nucleic acid vibrations at 787 cm^−1^ (ring breathing and O–P–O backbone stretching modes) and 1095 cm^−1^ (PO_2_^–^ groups), alongside protein-related peaks at 1005, 1251, 1455, and 1661 cm^−1^. On the basis of these spectral features, we assign it to nucleic acid–rich regions, such as cell nuclei.

Endmembers 4 (gray) and 5 (yellow) presented less conventional profiles, suggesting more complex biomolecular mixtures. Endmember 4 included peaks at 1021 cm^−1^ (glycogen), 1243 cm^−1^ (proteins), and 1671 cm^−1^ (proteins/lipids), and its spatial distribution overlapped with that of endmember 3.

Endmember 5 displayed a noisier spectrum, with higher abundance within the interior of the organoid. Prominent peaks were associated to proteins (574 cm^−1^, tryptophan/cytosine; 860 cm^−1^, C–C stretching; and 1215 cm^−1^, amide III) and carbohydrates (1024 cm^−1^, glycogen, and 1372 cm^−1^, CH deformation). Last, endmember 6 (magenta) showed a broadly distributed component, with bands related to proteins (1254, 1328, 1460, and 1649 cm^−1^) and nucleic acids (1098 cm^−1^).

Beyond 2D imaging, our approach also enables biochemical analysis in 3D. To illustrate this, we generated a volumetric rendering of the imaged organoid by overlaying the two derived components related to lipid-rich (endmember 2) and nucleic acid–rich (endmember 3) species as markers of membrane and nuclear regions, respectively. The reconstruction, displayed in Fig. 1H, allowed the visualization and analysis of the 3D organization and architecture of the imaged organoid tissue, including that of the present neural rosette (rosette lumen indicated with an arrow in Fig. 1D).

To confirm the assignment and interpretation of endmembers 2 and 3, we performed further spectral and spatial analysis, including comparison to reference spectra from pure solutions of DNA and POPC (1-palmitoyl-2-oleoyl-*sn*-glycero-3-phosphocholine, a phospholipid present in eukaryotic cell membranes) reported by LaLone *et al.* (*53*). The analysis showed that the two components exhibit preferential similarity to their corresponding references, with low spatial correlation across the organoid tissue (Fig. 1I and fig. S3). The derived organoid image using RS revealed characteristic morphology (see fluorescence microscopy image in Fig. 1C for reference) and was achieved without exogenous labeling or tissue sectioning. While fine-grained biomolecular assignments and spatial analysis are challenging at this resolution, this proof of concept demonstrates that our Raman-based imaging platform can effectively delineate organoid composition in situ and generate 2D and 3D morphochemical maps of key biochemical species in intact neural organoid samples in a fully label-free manner. Building on these results, we proceed to higher-resolution Raman imaging in the following sections.

### Experimental validation and optimization against fluorescence microscopy

To further validate that our method produces accurate morphochemical organoid reconstructions, we evaluated its performance against fluorescence microscopy and standard methods for hyperspectral RS analysis. To this end, defined tissue regions within two day-30 neural organoid cryosections were imaged using confocal Raman microspectroscopy, followed by immunostaining and fluorescence microscopy (Fig. 2A). Raman imaging was performed first to avoid potential signal interference from staining molecules. To ensure morphological diversity, we selected areas containing both distinct neural rosette structures and surrounding neural tissue for downstream imaging (Fig. 2B and figs. S4A and S5A). Raman imaging was performed over areas of 600 μm by 600 μm and 400 μm by 400 μm from the two respective sections, with a pixel size of 2 μm (figs. S4C and S5C). Subsequently, the organoid sections were stained for nuclei (DAPI), cell membranes (DiO), neural progenitor cells (SOX2), and neurons (TUJ1), and fluorescence microscopy images were taken to validate and correlate with organoid morphology predicted by RS (figs. S4B and S5B). To assess potential photodamage, we evaluated tissue integrity, Raman signal stability, and downstream fluorescence staining during/following Raman imaging. No detectable photodamage or imaging artefacts were observed under the acquisition conditions used (fig. S6).

**Fig. 2.**
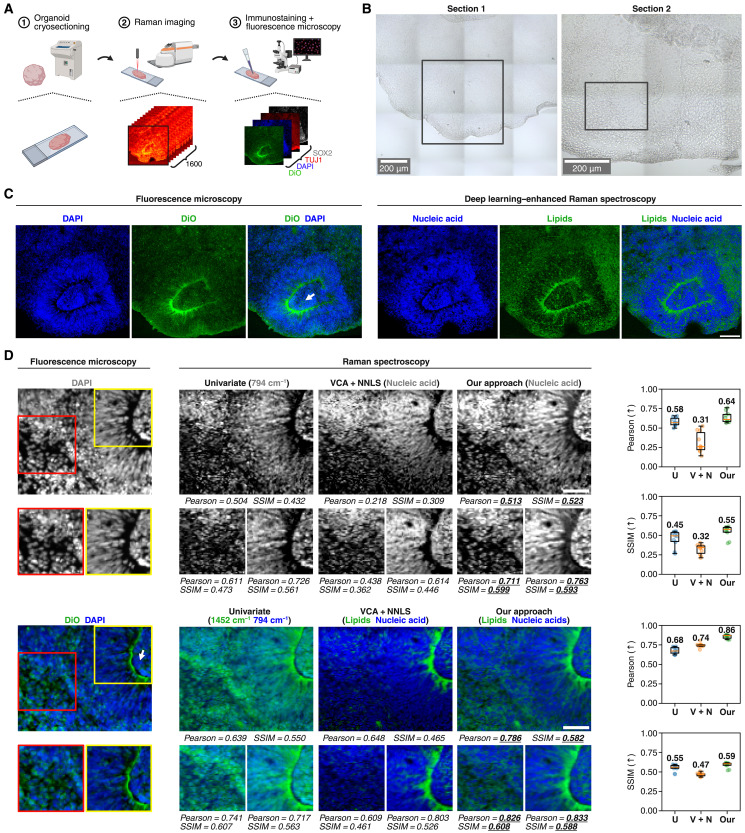
Deep learning–enhanced Raman microspectroscopy enables accurate morphochemical imaging of neural organoids as validated against fluorescence microscopy. (**A**) Schematic diagram of our validation strategy, which involved the collection of paired Raman imaging and fluorescence microscopy data from neural organoid cryosections. (**B**) Bright-field microscopy images of the two day-30 neural organoid cryosections imaged for validation. Marked areas show the regions used for validation in (C) and (D), respectively. Scale bars, 200 μm. (**C**) Imaging results on first organoid section. (left) Fluorescence microscopy images of DAPI (nuclei) and DiO (membranes). (right) RS abundance reconstructions of nucleic acid and lipids derived with our autoencoder pipeline. Arrow indicates the lumen of the present neural rosette. Scale bar, 100 μm. (**D**) Imaging results on second organoid section. (left) Fluorescence microscopy images of DAPI (nuclei) and DiO (membranes). (middle) RS abundance reconstructions of nucleic acid and lipids derived with (i) univariate analysis, (ii) unmixing analysis with VCA and NNLS, and (iii) our autoencoder pipeline. Similarity metrics, namely Pearson’s correlation coefficient (Pearson) and SSIM, were calculated relative to the corresponding downscaled fluorescence images. The highest similarity scores are indicated by underlined bold text. Arrow indicates the lumen of the present neural rosette. Scale bar, 50 μm. (right) Boxplots show Pearson and SSIM scores for 10 matched randomly selected crops (the same size as the red and yellow cutouts). Numbers indicate mean Pearson/SSIM scores. Our approach achieved the highest Pearson/SSIM scores for every individual crop. Paired Friedman tests, followed by Holm-corrected Wilcoxon signed-rank post hoc comparisons, indicated significant differences between methods (all adjusted *P* < 0.01). Raman data were acquired using the following parameters: pixel size = 2 μm in *x*-*y*, integration time = 0.5 s, laser power ≈ 48 mW, objective = 20×, NA 0.5. Created in BioRender. Georgiev, D. (2026) https://BioRender.com/5bdt9u2.

Using our autoencoder-based pipeline, we analyzed the acquired RS data from the first organoid section to infer its biochemical composition. Our method extracted four tissue-specific endmembers, which we associated with contributions from lipids, nucleic acids, and proteins (figs. S4, D to E, and S7). Figure 2C shows a side-by-side comparison between the predicted abundance maps of the two endmembers related to nucleic acid–rich and lipid-rich species and the corresponding fluorescence microscopy images of nuclei (DAPI) and cell membranes (DiO), respectively. We observed that our RS imaging pipeline accurately reconstructed individual nuclei, aligning with the DAPI staining across the imaged tissue (Fig. 2C, right, and fig. S4F). It also effectively captured important membrane features, including both fine cellular membranes and prominent structures such as the apical domain of the visible neural rosette. Qualitatively, we observed strong correspondence between the lipid-rich component obtained with RS and the DiO signal around the apical domain of the rosette and with the TUJ1 signal outside the rosette (fig. S4, B and E).

Having shown that our method accurately reconstructs key cellular components in cryosectioned neural organoid samples, we used the data collected from the second organoid section to perform a series of comparative analyses to test different versions of our autoencoder-based pipeline, including using different training losses (fig. S8) and endmember initialization procedures (fig. S9). Following this, we compared the unmixing performance of our developed approach against a range of standard chemometric methods for multivariate analysis. This included methods for dimensionality reduction [PCA (*60*, *61*) and nonnegative matrix factorization (*62*)], clustering [k-means clustering (*63*, *64*)], and hyperspectral unmixing [N-FINDR (*65*) and vertex component analysis (VCA) (*66*) for endmember identification, applied together with nonnegative least squares (NNLS) (*54*) for abundance estimation].

Our autoencoder-based pipeline derived six tissue-specific endmembers, which we characterized as lipid-, nucleic acid–, and protein-rich species (figs. S5D and S10). Visual examination of the estimated abundance maps revealed major cellular and subcellular features, including membranes, nuclei, cytoplasmic regions, and apical domains of neural rosettes (fig. S5E). Compared to standard methods, our approach offered improved robustness to artefacts and noise and produced more discriminative and interpretable abundance maps, enabling better-delineated imaging reconstruction of organoid morphology (fig. S11).

To quantitatively assess the performance of our autoencoder-based pipeline, we evaluated the image similarity between Raman-based reconstructions and fluorescence microscopy images in an overlapping organoid region (Fig. 2D). To this end, we spatially aligned both modalities and computed the Pearson’s correlation coefficient (Pearson) and structural similarity index measure (SSIM) (*67*) between RS-derived abundance maps of nuclei and membranes and fluorescence images of DAPI and DiO, respectively. We benchmarked the performance of our method against two conventional chemometric approaches as follows: (i) univariate analysis and (ii) multivariate hyperspectral unmixing using VCA and NNLS. To provide a favorable baseline, univariate analysis was performed using the intensity profiles at 794 and 1454 cm^−1^, which we found to be indicative of nuclear and lipid-rich membrane regions, respectively, using our autoencoder-based pipeline (fig. S5D). In practice, relevant peaks may be unknown or shifted because of experimental variations, calibration, preprocessing, and other factors.

Univariate analysis using the 794-cm^−1^ band yielded nuclear reconstructions that were generally aligned with the DAPI signal. However, visual inspection revealed irregular structures, which impeded the precise localization of individual nuclei (Fig. 2D, middle, first column). Major membrane structures, such as the apical domain of the imaged rosette, were also recovered via the 1454-cm^−1^ band, yet finer membrane features were not visually discernible, obscured by high background and nonspecific signal. This could be attributed to the overlap with vibrations of proteins in the same spectral range or nonspecific intensity variations due to acquisition or preprocessing settings, underscoring the need for more robust multivariate chemometric approaches (*68*). Multivariate unmixing analysis with VCA and NNLS showed low reconstruction accuracy, with further reduced Pearson and SSIM scores compared to univariate analysis for both nuclei and membranes (Fig. 2D, middle, second column). Visually, nuclear reconstructions were similarly nondistinct, with poorly defined nuclei. Moreover, while major membrane structures like the apical domain of the present rosette were recovered with higher specificity, finer membrane features were consistently undetected.

By contrast, as shown in Fig. 2D (middle, third column), our autoencoder-based pipeline consistently outperformed univariate analysis and VCA + NNLS, yielding higher reconstruction accuracy for both nuclear and membrane-associated structures—quantitatively, with superior Pearson and SSIM scores, and qualitatively, with more interpretable and biologically relevant output. For nuclear reconstructions (Fig. 2D, top row), Pearson correlation increased from 0.504 (univariate analysis) and 0.218 (VCA + NNLS) to 0.513 with our approach, alongside improvements in SSIM from 0.432 and 0.309 to 0.523. Qualitatively, our method recovered better-defined nuclei with sharper nuclear boundaries and lower nonspecific signal. Incorporating membrane features (Fig. 2D, bottom row) further highlighted enhanced reconstruction, with Pearson correlation rising from 0.639 and 0.648 to 0.786, and SSIM values rising from 0.550 and 0.465 to 0.582. Visually, our method produced more clearly delineated membrane structures, revealing both large-scale features such as apical domains and individual cellular membranes. These improvements were consistent across two ROIs with distinct tissue morphologies (55 by 55 pixels), including general tissue and neural rosette regions (Fig. 2D, small crops).

To further quantify robustness, we evaluated Pearson correlation and SSIM on 10 matched randomly selected crops of the same size (Fig. 2D, right). Our method achieved the highest Pearson and SSIM scores in every crop across all four analyses, improving the mean Pearson/SSIM by 0.04 to 0.11 over the strongest baseline (≈7 to 20% relative). Paired Friedman tests, followed by Holm-corrected Wilcoxon signed-rank post hoc comparisons, indicated significant differences between methods at the ROI level in all cases (all adjusted *P* < 0.01).

Last, we further assessed model robustness and run-to-run variability. Our analyses demonstrated that the pipeline is stable across runs, consistently recovering meaningful endmembers and abundance maps (fig. S12), and is robust to small variations in the number of endmembers extracted (fig. S13). We also showed that our pipeline yields consistent and reproducible endmembers across all datasets and acquisition settings used in this study (fig. S14). Furthermore, our approach demonstrated improved robustness to experimental noise, maintaining consistent reconstruction quality under reduced denoising conditions. In comparison, the performance of conventional methods deteriorated markedly, yielding noisier, less interpretable spectral components, and abundance profiles, or failing to converge to a solution during unmixing altogether (fig. S15).

### Biochemical imaging of intact neural organoids at subcellular resolution

Having demonstrated that our method achieves accurate subcellular imaging of 2D cryosectioned neural organoid tissue, we next performed higher-resolution Raman imaging in intact 3D neural organoids. The ability to resolve and visualize subcellular structures in intact organoid tissues in their native environment is critical for interrogating important neurodevelopmental processes, such as nuclear positioning, polarity establishment, and lumen formation, and studying their effect on brain tissue organization, architecture, and function. Imaging in intact samples, however, is inherently more challenging because of optical effects such as increased scattering and depth-dependent signal attenuation. In RS, these factors can degrade unmixing fidelity and hinder the extraction of meaningful biochemical features.

To examine the effectiveness of our pipeline beyond tissue sections, we acquired a larger 400-by-346 Raman imaging scan of a tissue region within an intact neural organoid, taken with 1-μm pixel size from an intact day-10 neural organoid sample (Fig. 3A). Using our Raman imaging pipeline, we analyzed the data and identified six tissue-specific components. The spectral endmembers and corresponding abundance maps are presented in Fig. 3 (B and C, respectively) (extended data in fig. S16). Endmember 1 (red) exhibited a lipid-rich profile with prominent peaks at 1077 cm^–1^ (C–C and C–O stretching, phospholipids), 1305, 1448, and 1657 cm^–1^. Its abundance map outlined membrane-like structures, along with more confined bright spots resembling lipid droplets. Endmember 2 (gray) was characterized by vibrations associated with both protein and lipid species, with peaks at 1000, 1124 cm^−1^ (C–C and C–N stretching), 1249, 1311, 1454, and 1654 cm^−1^. Its abundance was observed in regions partially overlapping with membranes, as well as in isolated domains, possibly indicating cytoplasmic or extracellular matrix compartments. Endmember 3 (blue) was marked by strong nucleic acid bands at 790 and 1095 cm^−1^, alongside protein-associated bands at 1005, 1249, 1328, 1455, and 1659 cm^−1^. As in previous analyses, the abundance map revealed well-defined spherical compartments consistent with individual cell nuclei. Endmember 4 (green) presented a distinct protein-rich signature with peaks at 1005, 1251, 1454, and 1662 cm^–1^, along with vibrations associated with ring breathing modes in nucleic acid bases at 1573 cm^–1^. The abundance map highlighted cellular-level compartments resembling cytoplasmic regions, as well as small punctate structures within nuclei with high signal intensity, potentially corresponding to biomolecular condensates, such as nucleoli. Endmember 5 (yellow) displayed a mixed profile comprising bands linked to proteins (1259, 1326, 1453, and 1662 cm^−1^) and nucleic acid (1095 cm^−1^). This component appeared more diffusely distributed, with substantial spatial overlap with endmember 3. Endmember 6 (purple) presented a noisier signature that contained key carbohydrate bands at 493 cm^−1^ (glycogen), 850 cm^−1^ (C–O–C skeletal mode, glycogen), 938 cm^−1^ (C–C stretching), and 1103 cm^−1^ (C–O and C–O–C stretching), along with a peak at 754 cm^−1^ associated with cytochrome *c*. The abundance overlapped with endmember 4, potentially reflecting glycoproteins within nuclear substructures and condensates, and glycogen stores in perinuclear regions and the cytoplasm. The proposed biochemical identities of endmembers 1, 3, 4, and 6 were further validated by comparison with reference spectra of POPC, DNA, protein, and glycogen reported by LaLone *et al.* (*53*), respectively, with each endmember exhibiting characteristic Raman bands corresponding to its assigned biomolecular class (fig. S17).

**Fig. 3.**
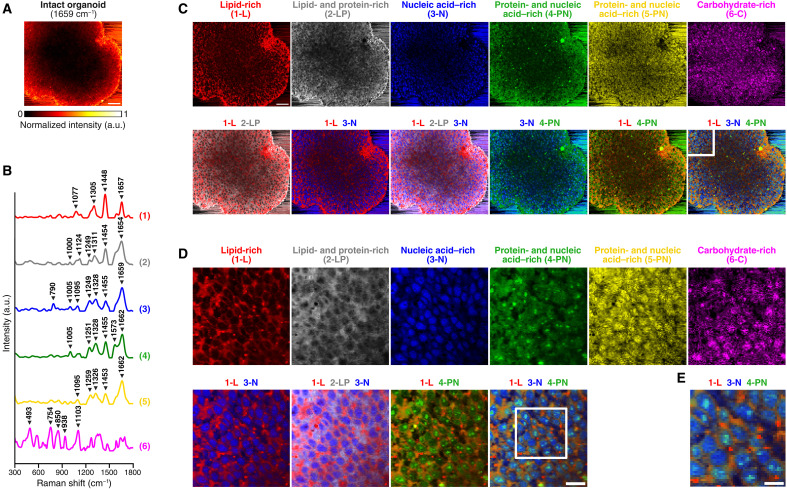
Biochemical imaging of intact neural organoids at subcellular resolution. (**A**) Snapshot of collected Raman imaging data from an intact day-10 neural organoid sample, showing the intensity profile at the 1659 cm^–1^ band (proteins and lipids). Scale bar, 50 μm. (**B** to **E**) Results of hyperspectral unmixing analysis. (B) Derived tissue-specific endmember signatures. Endmembers scaled for visualization purposes (maximum intensity set to 1). (C) Corresponding abundance maps. Scale bar, 50 μm. (D) Zoomed-in abundance images of a selected area [area marked in (C) bottom right]. Scale bar, 20 μm. (E) Further zoomed-in abundance image [area marked in (D)]. Scale bar, 10 μm. Raman data were acquired using the following parameters: pixel size = 1 μm in *x*-*y*, integration time = 0.5 s, laser power ≈ 32 mW, objective = 63×, NA 1. Created in BioRender. Georgiev, D. (2026) https://BioRender.com/vfwohcm.

To further inspect the spatial reconstructions produced by our imaging pipeline, we examined magnified fields from selected regions within the imaged organoid tissue (Fig. 3, D and E). The resulting reconstructions revealed consistent, well-defined cellular features, which delineated individual cells and subcellular structures resembling nuclei, cytoplasmic regions, cellular membranes, and nuclear condensates. These results demonstrate the effectiveness of our imaging platform in intact samples across scales and resolutions, allowing us to resolve complex biochemical heterogeneity and interrogate key morphological features at cellular and subcellular scales in a single, label-free imaging modality.

### Volumetric in situ imaging of a neural rosette within an intact neural organoid

Next, we extended our subcellular imaging analysis to 3D and used our pipeline to perform volumetric in situ imaging of a neural rosette structure within an intact neural organoid. Neural rosettes are important organizational units during neural organoid development, which recapitulate key aspects of neural tube formation in the embryonic central nervous system (*69*). They typically form within the first 1 to 2 weeks of neural induction and appear as radially organized, layered structures composed primarily of neuroepithelial cells at early stages. As development progresses, these progenitor cells give rise to differentiated neural lineages, including neurons and glial cells. To illustrate this, we performed fluorescence microscopy of a day-20 neural organoid tissue section, with markers for nuclei (DAPI), neurons (MAP2), neural progenitor cells (SOX2), and apical domain (PKCζ). The resulting images, shown in Fig. 4A, revealed multiple neural rosettes with characteristic radial structure. We observed an abundance of neural progenitor cells densely organized within the rosettes, with surrounding layers of differentiating neurons.

**Fig. 4.**
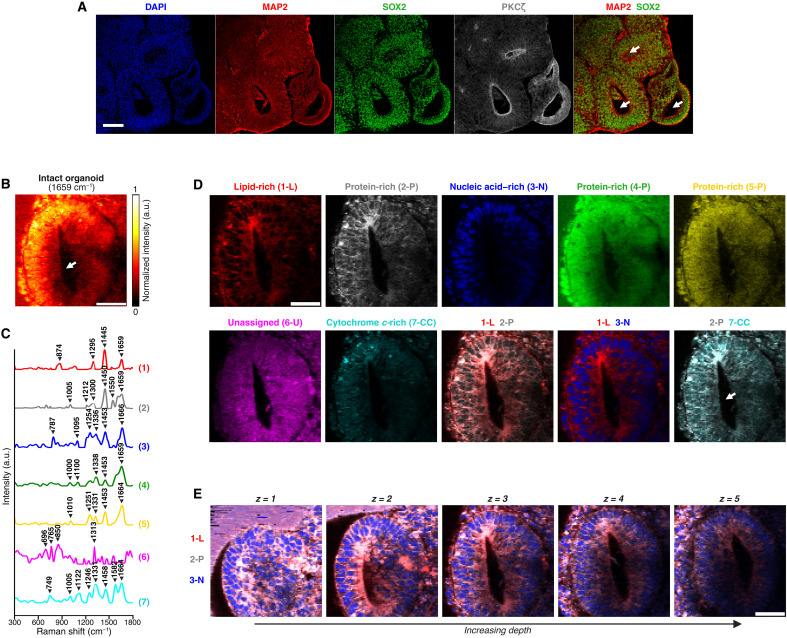
Volumetric in situ biochemical imaging of a neural rosette within an intact neural organoid. (**A**) Fluorescence microscopy images of a day-20 neural organoid tissue section, showing characteristic neural rosette structures, with markers for nuclei (DAPI), neurons (MAP2), neural progenitor cells (SOX2), and apical domain (PKCζ). Arrows indicate the lumens of developed neural rosettes. Scale bar, 100 μm. (**B**) Snapshot of collected volumetric Raman imaging data from an intact day-20 neural organoid sample (selected *z* layer), showing the intensity profile at the 1659 cm^−1^ band (proteins and lipids). Arrow indicates the lumen of the present neural rosette. Scale bar, 50 μm. (**C** to **E**) Results of hyperspectral unmixing analysis. (C) Derived tissue-specific endmember signatures. Endmembers scaled for visualization purposes (maximum intensity set to 1). (D) Corresponding abundance maps for a representative *z* layer. Arrow indicates the lumen of the present neural rosette, as also shown in (B). Scale bar, 50 μm. (E) Image reconstructions of selected endmember components at different depths in *z*. Scale bar, 50 μm. Raman data were acquired using the following parameters: pixel size = 2 μm in *x*-*y* and 10 μm in *z*, integration time = 0.5 s, laser power ≈ 32 mW, objective = 63×, NA 1.Created in BioRender. Georgiev, D. (2026) https://BioRender.com/3umu929.

Following this, we investigated whether our Raman-based imaging platform could be used to visualize and analyze the structure of a neural rosette without the need for tissue sectioning and exogenous labeling. To this end, we acquired a volumetric RS scan of a neural rosette within an intact day-20 neural organoid, consisting of five imaging slices in *z* (organoid depth), with each slice measuring 95 by 95 pixels with a pixel size of 2 μm. Figure 4B displays the intensity profile at the 1659 cm^−1^ peak (proteins and lipids) of one of the collected layers, outlining a characteristic neural rosette structure [see Fig. 4A for reference in 2D or Masselink *et al.* (*70*) for fluorescence microscopy of a neural rosette in intact tissue-cleared neural organoid]. Our autoencoder-based unmixing analysis yielded six tissue-specific components (Fig. 4, C and D, and extended data in fig. S18). Endmember 1 (red) presented a lipid-rich profile with prominent peaks at 874 cm^−1^ (phospholipids), 1295 cm^−1^ (CH_2_ deformation), 1445, and 1659 cm^−1^. The corresponding abundance map highlighted structures resembling cellular membranes and lipid bodies. Endmember 2 (gray) contained features associated with proteins and lipids, with peaks at 1005, 1300, 1450, 1550 cm^−1^ (C=C stretching, tryptophan), and 1659 cm^−1^. Its signal overlapped with that of endmember 1, appearing in membrane-like regions but also extended into the cell interior, possibly indicating cytoplasmic and structural proteins. Endmember 3 (blue) showed a distinct nuclear signature, with nucleic acid–associated vibrations at 787 and 1095 cm^−1^, along protein bands at 1254, 1336, 1453, and 1666 cm^−1^. This component exhibited strong nuclear specificity, outlining well-defined spherical compartments consistent with individual cell nuclei. Endmembers 4 (green) and 5 (yellow) shared similar spectral features dominated by protein-related peaks around ∼1005, ∼1100, ∼1255, ∼1335, ∼1453, and ∼1660 cm^−1^, suggesting general protein composition. Endmember 6 (magenta) displayed a less conventional, noisier profile with peaks at 696 cm^−1^ (potentially related to cholesterol), 765 cm^−1^ (pyrimidine ring breathing in nucleic acids), 850 cm^−1^ (C–O–C skeletal mode in carbohydrates, glycogen), and 1313 cm^−1^ (CH_2_/CH_3_ twisting in lipids and collagens, or guanine in nucleic acid). This component was generally broadly distributed, with some confined regions with higher abundance intensity. Last, endmember 7 (cyan) showed a signature well aligned with mitochondria, featuring peaks associated with cytochrome *c* at 749, 1122, and 1582 cm^−1^, alongside protein-related bands at 1005, 124, 1331, 1458, and 1664 cm^−1^. The derived endmember showed close correspondence to a reference spectrum from a pure solution of cytochrome *c* reported by LaLone *et al.* (*53*), particularly at the canonical cytochrome *c* bands at 749, 1122, and 1582 cm^−1^ (fig. S17). This component was observed in cytoplasmic regions, in proximity to nuclei, consistent with intracellular localization of mitochondria.

To examine the 3D structure of the scanned rosette, we overlaid the abundance maps of the derived lipid-rich (endmember 1), protein-rich (endmember 2), and nucleic acid–rich (endmember 3) components across all five *z* layers. The resulting images, presented in Fig. 4E, demonstrated that our imaging pipeline can effectively reconstruct key subcellular features—such as individual cellular membranes and nuclei—across all five imaging scans, enabling the interrogation of cellular architecture and organization within the intact neural rosette in both 2D and 3D. However, note that reconstructions progressively decrease in intensity with depth due to depth-dependent signal attenuation in intact samples. We explore normalization as a strategy for mitigating this effect in the next section.

### Tracing changes in biochemical composition during early neurodevelopment in intact neural organoids

During maturation, neural organoids recapitulate key stages of early human brain development, including the formation of distinct progenitor zones and the emergence of layered neural architectures (*11*, *12*, *71*, *72*). On the basis of prior brain organoid studies (*7*, *73*, *74*) and a recent report describing the temporal progression of marker expression in neural organoids (*75*), we selected five representative time points for RS analysis—namely, days 5, 10, 20, 30, and 40—which capture key stages of neural organoid development. Day 5 corresponds to an early preneural induction stage dominated by pluripotent stem cells; day 10 marks the emergence of neural progenitor populations following the transition from stem cell to neural induction medium at day 5; and days 20, 30, and 40 represent progressively later stages of neuronal differentiation, during which neural progenitor markers plateau or decline while neuronal markers gradually increase over time.

To investigate the biochemical dynamics accompanying tissue differentiation and organization, we applied our imaging pipeline to trace changes in organoid composition during neural organoid development. To this end, we acquired Raman imaging data from organoid samples at each of the five aforementioned developmental time points—days 5, 10, 20, 30, and 40 (Fig. 5A). In total, we collected *72* Raman imaging scans from organoids grown in three independent culture batches, with scan numbers ranging between 11 and 17 per time point (fig. S19A). Each scan represented a 20-by-160 image across the outermost tissue layers, which normally contain the most metabolically active and viable cells (*76*, *77*). Measurements were acquired with a lateral pixel size of 5 μm in the *x*-*y* plane and an axial step size of 10 μm along the *z* axis, thereby imaging up to 800 μm across and 200 μm deep into the organoid samples. To mitigate depth-dependent signal attenuation, we applied pixelwise (vector) normalization in this analysis rather than the global strategy used in previous sections. This approach provided substantially improved signal recovery at depth while preserving relative abundance trends across experimental groups (fig. S20). However, we emphasize that pixelwise normalization removes absolute intensity information and is applied here specifically for analyses focused on relative trends across time point groups, with comparisons restricted to comparable fields of view across organoid samples.

**Fig. 5.**
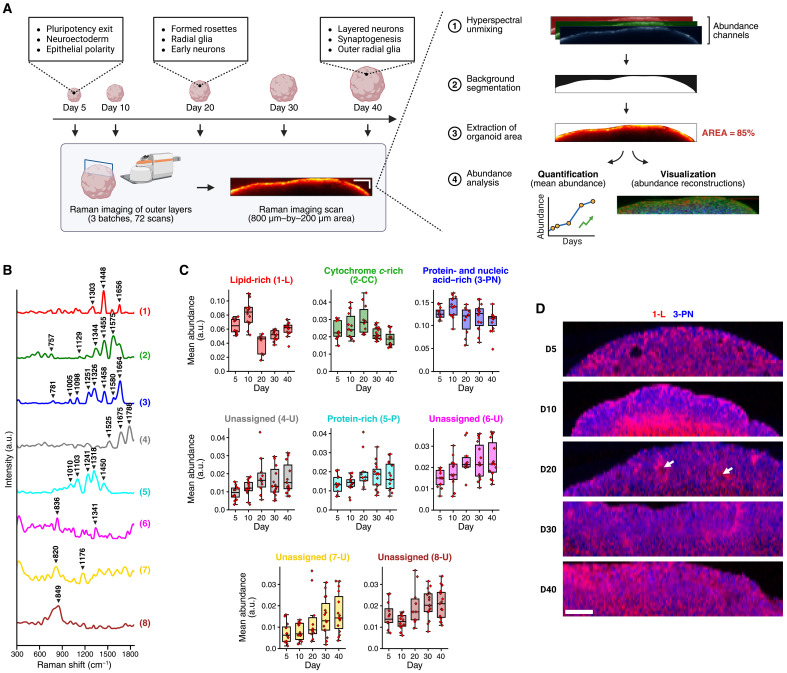
Tracing changes in biochemical composition during early neurodevelopment in intact neural organoids. (**A**) Schematic diagram of our time point study, depicting data acquisition (left) and analysis (right). Scale bars, 100 μm. (**B** to **D**) Results of hyperspectral unmixing analysis. (B) Derived tissue-specific endmember signatures. Endmembers scaled for visualization purposes (maximum intensity set to 1). (C) Quantification of the corresponding abundances over time. Sample sizes: day 5 (*n* = 12), day 10 (*n* = 14), day 20 (*n* = 10), day 30 (*n* = 15), and day 40 (*n* = 15) from *N* = 3 independent organoid culture batches. (D) Abundance reconstructions of selected organoid samples across the five time point groups. Arrows indicate structures suggestive of rosette-like organization based on morphology and developmental timing. Scale bar, 100 μm. Raman data were acquired using the following parameters: pixel size = 5 μm in *x*-*y* and 10 μm in *z*, integration time = 0.5 s, laser power ≈ 32 mW, objective = 63×, NA 1. Created in BioRender. Georgiev, D. (2026) https://BioRender.com/w6o9ike.

After preprocessing and normalization, hyperspectral unmixing analysis was performed on the combined timepoint dataset (i.e., all scans pooled and analyzed jointly) to ensure that a single shared set of endmember spectra was learned and that abundances are directly comparable across time points. The analysis yielded eight tissue-specific endmember species (Fig. 5B and extended data in fig. S21). The corresponding abundance profiles were analyzed to examine relative biochemical trends across developmental groups. Figure 5C displays the mean abundance of each component across time points, after segmentation and background removal, and area normalization (figs. S19B and S22).

Endmember 1 (red) exhibited a distinct lipid-rich spectral profile, characterized by prominent peaks at 1303, 1448, and 1656 cm^−1^, and appeared broadly distributed with high abundance across all organoids and time points (fig. S23A). Still, distinct spatial and temporal variations in its distribution across time points were observed. On day 5, when differentiation has not yet begun and a relatively uniform population of pluripotent stem cells is expected, the signal appeared homogeneous, with slightly elevated intensity in small compartments near the outer layers. By day 10, when neural stem/progenitor cell populations expand and pluripotent stem cell populations decline following neural induction, the signal intensity increased markedly, particularly around the inner layers and surface. The increase in abundance may reflect shifts in lipid metabolism during early neurogenesis, in line with previous findings that lipid accumulation is essential for neural stem/progenitor cell activity and proliferation (*78*). At day 20, a developmental stage associated with neural rosette formation and early neuronal differentiation, a sharp decline in signal was observed, together with the emergence of discrete, localized regions of elevated intensity, possibly corresponding to developed neural rosette structures. During later stages (days 30 and 40), when neuronal differentiation and maturation become more prominent, the signal progressively recovered, once again displaying a more uniform and widespread distribution. This gradual recovery may be linked to the expansion and functional maturation of neuronal populations, which rely on lipids not only as essential structural components of membranes but also as signaling molecules with key regulatory functions (*79*, *80*). This is further supported by our observation that lipid-rich endmembers are well co-localized with TUJ1-positive regions—a marker of neurons (fig. S4).

In contrast, endmember 2 (green) presented a mixed spectral profile, with peaks linked to cytochrome *c* (757, 1129, and 1575 cm^−1^) and proteins (1344 and 1455 cm^−1^). This signature may suggest metabolically active regions enriched in mitochondria (*81*). Its abundance profile followed an inverted U-shaped trajectory. It gradually increased from day 5 to day 20, possibly reflecting rising metabolic demand during early neuronal differentiation and a metabolic shift from glycolysis toward oxidative phosphorylation as stem cells and neural progenitors mature into neurons, accompanied by increased cytochrome *c* expression (*82*, *83*). At later time points (days 30 and 40), associated with neuronal maturation and reduced proliferative activity, the abundance declined.

Endmember 3 (blue) comprised vibrational features associated with nucleic acids (781, 1098, and 1580 cm^−1^) and proteins (1005, 1251, 1326, 1458, and 1664 cm^−1^), likely corresponding to cytoplasmic and nuclear regions. Its abundance was generally stable across developmental stages, with a slight downward trend. A similar progression was reported by Bruno *et al.* (*52*), who observed a gradual decrease in nucleic acid and protein features in maturing cortical organoids. Visually, this component was highly abundant across all organoid samples, with a generally uniform distribution throughout organoids, and slightly elevated intensity in the outer tissue layers (fig. S23B).

Endmembers 4 to 8 exhibited less conventional spectral profiles, with lower relative abundance predominantly present in deeper organoid regions (fig. S21C). They shared similar developmental trajectories, each showing a gradual increase in abundance over time. While prominent peaks within these endmembers allow for tentative biochemical assignments, further validation is required to determine whether these components reflect representative biological features or traces arising from higher noise levels in deeper tissue due to depth-dependent signal attenuation. To reflect this uncertainty, these five endmembers are left unassigned in the analysis. Endmember 4 (gray) featured two bigger peaks at 1675 and 1788 cm^−1^, likely attributable to protein species, and a smaller peak 1525 cm^−1^, potentially indicating contributions from carotenoids (C–C and C=C stretching). Endmember 5 (cyan) was characterized by distinct protein-related peaks at 1010, 1103, 1241, 1318, and 1450 cm^−1^. Endmembers 6 (magenta) and *7* (yellow) exhibited noisier profiles, featuring multiple, less-defined peaks. Some of the more pronounced vibrations included peaks at 820, 836, 1176, and 1341 cm^−1^, all related to proteins. Last, endmember 8 (brown) showed a single dominant peak at 849 cm^−1^, potentially corresponding to the C–O–C skeletal vibrations of saccharides. Bruno *et al.* (*52*) reported a similar peak around 850 cm^−1^ as a prominent predictive marker of cortical organoid maturation, showing a progressive increase between weeks 6 and 20. The authors attributed this feature to glycogen and associated its rise with metabolic changes accompanying neuronal differentiation. Statistical testing showed significant differences at the ROI level across the first three components, which were associated with lipid-rich, cytochrome *c*-rich, and protein- and nucleic acid–rich regions (endmembers 1, 2 and 3, respectively), as well as for endmembers 6 and 8, which were left unassigned (fig. S24).

To illustrate the dynamic morphochemical composition of neural organoids during early neurodevelopment, we further generated spatial reconstructions of selected biochemical components across organoid samples at different developmental stages. Figure 5D displays reconstructions of representative samples from each of the five time points, highlighting the two selected components associated with lipids, and proteins and nucleic acids, respectively (see fig. S21C for other components). These visualizations enabled us to trace the evolving spatial organization and architecture of neural organoids across their development, providing insights into neural organoid maturation and further supporting the quantitative trends observed in Fig. 5C. In particular, structures suggestive of rosette-like organization were observed at the day-20 time point (marked with white arrows in Fig. 5D), based on morphology and developmental timing. Further validation combining volumetric Raman imaging and fluorescence microscopy would be essential to more conclusively characterize developmental structures and trajectories obtained with RS in intact organoid samples. Together, our results provide proof of concept for the feasibility of label-free, RS-based biochemical analysis of spatiotemporal dynamics in intact neural organoids during development.

## DISCUSSION

We have developed here a method for noninvasive, label-free organoid imaging based on Raman microspectroscopy combined with unsupervised autoencoder-based hyperspectral analysis. We showed that our approach is effective at discerning key biochemical components in unlabeled RS measurements, enabling 2D and 3D visualizations of structural and morphological features in both cryosectioned and intact neural organoids and at different scales and resolutions. Using our imaging platform, we studied structural and biochemical features of early neurodevelopment in neural organoids and demonstrated the ability to image neural rosettes within intact organoid samples in 3D. Furthermore, we investigated changes in the biomolecular composition of intact neural organoids during early developmental stages, observing distinct spatiotemporal dynamics as organoids mature in major biomolecular classes, including lipids, proteins, and nucleic acids.

By eliminating the need for destructive preparation steps and external labels, our workflow offers simpler sample preparation that does not compromise organoid integrity, with potential for live-organoid analysis. This opens avenues for research into early neurodevelopment but also in developmental diseases and drug discovery, where our approach could allow studying disease etiology in neural organoid disease models or visualizing drug distribution in situ. We note that our pipeline can also be applied to other types of samples, including other types of organoids and tissues. Beyond imaging, it can also be used as a feature extraction technique in RS for downstream applications—e.g., organoid phenotyping, disease classification, and drug response modeling.

In this study, we used spontaneous RS to validate our pipeline under stable, reproducible conditions using fixed neural organoids. Although inherently time-consuming, spontaneous RS offers high spectral resolution and chemical specificity and is more broadly accessible than faster, more advanced RS modalities, making it well suited for method development and benchmarking. However, we note that our unmixing analysis framework is also compatible with emerging broadband coherent Raman scattering modalities (*84*), which support video-rate imaging over wide spectral ranges. For example, Vernuccio *et al.* (*85*) recently reported a microscopy system based on coherent anti-Stokes Raman scattering capable of full-spectrum acquisitions over large fields of view at millisecond dwell times, combined with standard methods for unmixing (N-FINDR and NNLS). In this context, spectral unmixing remains a key step in the analysis pipeline, and future work integrating our method with more advanced RS techniques could allow fast, high-throughput analysis of biological specimens with improved chemical accuracy—a major step toward real-time, label-free imaging of live organoids. However, further advances in organoid culture, real-time environmental control, sample holders, and high-speed acquisition protocols are needed to standardize measurement conditions and bridge the gap between fixed and live organoid systems.

Last, we note that although derived endmembers were characterized using established Raman bands, their biochemical interpretation remains post hoc. Hence, some peak assignments remain partially ambiguous because of the biochemical complexity of neural organoids and depth-dependent signal attenuation. Future work integrating Raman imaging with complementary imaging modalities could further improve qualitative and quantitative interpretation. This could also enable more comprehensive studies into model robustness and reliability across runs, datasets, and hyperparameters, which remains a challenge in blind, data-driven unmixing. Nonetheless, our work demonstrates the feasibility of label-free, RS-based biochemical imaging and analysis in complex, 3D neural organoid systems.

## MATERIALS AND METHODS

### Organoid culture and preparation

Human embryonic stem cells (WAe009–A, RRID:CVCL_9773, WiCell) were cultured feeder-free on hES-qualified Matrigel (354277, Corning)–coated plates in a 5% CO_2_ incubator at 37°C. mTeSR1 medium (STEMCELL Technologies) was used for daily cell maintenance according to the manufacturer’s instructions. To generate neural organoids, cells were dissociated into single cells using Accutase (STEMCELL Technologies), resuspended in mTeSR1 supplemented with 50 μm of Y-27632 (S1049, Selleck Chemicals) and 1% (v/v) penicillin-streptomycin (15140122, Thermo Fisher Scientific), and then added to an ultralow-binding 96-well plate (Corning) at 9000 cells/150 μm per well. On day 3, 100 μm of the media was removed and 150 μm fresh mTeSR1 containing 1% (v/v) penicillin-streptomycin was added. From day 5 onward, the media were changed to neural induction media, composed of Dulbecco’s modified Eagle’s medium (DMEM)/F12 (Thermo Fisher Scientific) supplemented with heparin (1 μm/ml; Sigma-Aldrich), 1% (v/v) penicillin-streptomycin, 1% (v/v) GlutaMAX (Thermo Fisher Scientific), 1% (v/v) N2 supplement (Thermo Fisher Scientific), and 1% (v/v) MEM Non-Essential Amino Acids (MEM-NEAA) (Thermo Fisher Scientific). On day 10, organoids were embedded in Matrigel (354234, Corning) and transferred to 10-cm petri dishes. From day 12 to day 18, the culture medium was switched to Improved-A medium consisting of a 1:1 mixture of DMEM/F12 and Neurobasal medium (Thermo Fisher Scientific) supplemented with 1% (v/v) penicillin-streptomycin, 1% (v/v) B27 supplement without vitamin A (Thermo Fisher Scientific), 1% (v/v) GlutaMAX, 0.5% (v/v) N2 supplement, 1/4000 (v/v) insulin (I9278, Sigma-Aldrich), 0.5% (v/v) MEM-NEAA, and sodium bicarbonate (1 mg/ml). Organoids were fed with fresh Improved-A medium every 3 days—i.e., on days 12 and 15. From day 18 onward, the petri dishes were placed on an orbital shaker, and the culture medium was replaced with Improved+A medium consisting of a 1:1 mixture of DMEM/F12 and Neurobasal medium (Thermo Fisher Scientific) supplemented with 1% (v/v) penicillin-streptomycin, 2% (v/v) B27 supplement with vitamin A (Thermo Fisher Scientific), 1% (v/v) GlutaMAX, 0.5% (v/v) N2 supplement, 1/4000 (v/v) insulin (I9278, Sigma-Aldrich), 0.5% (v/v) MEM-NEAA, sodium bicarbonate (1 mg/ml), and vitamin C (0.07 mg/ml).

Organoids were fixed at days 5, 10, 20, 30, and 40 in 4% (w/v) paraformaldehyde overnight at 4°C and then rinsed three times with phosphate-buffered saline (PBS) (Thermo Fisher Scientific). Fixed organoids were then immobilized on a 1% (w/v) agarose layer and immersed in PBS for Raman imaging.

To prepare organoid slices for Raman imaging or immunostaining, organoid tissues were cryosectioned. Briefly, fixed organoid tissues were first incubated in 30% sucrose at 4°C for a day on a roller mixer and then transferred into Tissue-Tek O.C.T. compound (Sakura Finetek USA), gently swirled, and incubated for 5 min. The tissues were then frozen in a cryomold filled with Tissue-Tek O.C.T. compound to form cryoblocks containing the frozen organoids. For the two cryosections prepared for RS imaging, this was achieved using the technique for high-throughput cryoprocessing previously reported by Reumann *et al.* (*75*). The cryoblocks were subsequently sectioned into 20-μm slices using a CryoStar NX70 Thermo Cryostat and collected on positively charged cryoslides. The tissue sections were dried for at least 3 hours before being stored at −20°C or used for downstream analysis (fluorescence microscopy and Raman microspectroscopy). Tissue sections were immersed in Ultra-Pure deoxyribonuclease/ribonuclease-free distilled water (Invitrogen) for Raman imaging.

### Immunofluorescence staining and microscopy

For immunostaining, microscopy slices were washed in PBS for 5 min to wash off residual O.C.T. and then permeabilized and blocked in PBS containing 0.3% (v/v) Triton X-100 (Thermo Fisher Scientific) and 5% (w/v) bovine serum albumin (BSA, Thermo Fisher Scientific) for 0.5 to 1.5 hours at room temperature. Primary and secondary antibodies were diluted in PBS containing 0.1% (v/v) Triton X-100 and 5% (w/v) BSA. Primary antibody solutions were incubated with the slices overnight at 4°C, followed by three rinses in PBS, and three washes with PBS-T (PBS + 0.01% TX100) for 10 to 15 min on a slow rotating orbital shaker at room temperature. Secondary antibody solutions were then added and incubated for 2 hours at room temperature. Extended information about the primary and secondary antibodies used for immunostaining is provided in tables S1 and S2, respectively. Cell membranes were labeled using DiO (BT60011, Biotium)—a fluorescent lipophilic dye—which was added to the secondary antibody solution. DAPI (Thermo Fisher Scientific), diluted 1:1000 in PBS, was used to stain nuclei for 7 min. After this, the slides were rinsed three times in PBS, and washed twice in PBS-T and once in PBS for 10 to 15 min on a slowly rotating orbital shaker. Stained organoid slices were then mounted using fluorescent mounting media (S302380-2, Agilent DAKO) or FluoroSave (Millipore). Fluorescent images were acquired using a Leica SP8 confocal microscope and a Nikon TI-2 wide-field fluorescent microscope, and processed in FIJI.

### Raman microspectroscopy

RS measurements were performed using a confocal Raman microscope (alpha300 R, WITec GmbH) equipped with a 532-nm solid-state laser (WITec GmbH). Laser power at the sample plane was set to about 48 mW for organoid cryosections or 32 mW for intact samples. Backscattered light was collected through a water immersion objective – 20× [Zeiss W N-Achroplan, numerical aperture (NA) = 0.5] for organoid cryosections or 63× (Zeiss W Plan-Apochromat, NA = 1) for intact samples and delivered via a 10-μm single-mode silica fiber to an imaging spectrograph (Newton, Andor Technology Ltd.) with a 600–groove/mm grating and a thermoelectrically cooled charge-coupled device detector (−60°C). The system provided a spectral resolution of ∼2 to 3 cm^−1^ over the wavenumber range 0 to 3670 cm^−1^. Spectra were acquired with pixel sizes in *x* and *y* and step sizes in *z* ranging from 1 to 10 μm, and an integration time of 0.5 s.

### Spectral preprocessing of Raman imaging data

Acquired RS measurements were exported as MATLAB files using the WITec Project FIVE Suite software and loaded in Python using RamanSPy (*56*). Following this, each scan was individually preprocessed using RamanSPy as follows: (i) spectral cropping whereby wavenumbers less than 300 cm^−1^ were removed; (ii) cosmic rays removal using the algorithm reported by Whitaker and Hayes (*86*), with a kernel size *m* = 3 and *z*-value threshold = 6; (iii) denoising with a third-order Whittaker smoother (*87*) with λ = 10^3^; (iv) baseline correction with second-order asymmetric least squares (*88*) with λ = 10^5^; and (v) vector normalization. Normalization was applied on a pixel-wise basis for our time point data to account for depth-dependent signal attenuation. For the rest of the data, global vector normalization was performed where intensity values were divided by the $\ell^{2}$-norm of the spectrum with the highest norm in the given scan. In addition, wavenumber calibration was performed on our cryosection scans by calculating the median position of the laser peak (Rayleigh scattering) and setting it to zero.

### Hyperspectral unmixing analysis

Chemometric analysis was performed using a custom autoencoder neural network model designed for blind hyperspectral unmixing. In this context, Raman measurements $x_{i}\in {\mathbb{R}}^{b}$ are treated as mixtures $x_{i}=ℱ\left(M,\alpha_{i}\right)$ of a set of *n* unknown endmember components $M\in {\mathbb{R}}^{n\times b}$ based on their relative abundances $\alpha_{i}\in {\mathbb{R}}^{n}$ in a given measurement $x_{i}$. Blind unmixing aims to decompose a set of Raman measurements $X\in {\mathbb{R}}^{m\times b}$ (i.e., a given scan) into endmember components M and their relative abundances $A\in {\mathbb{R}}^{m\times n}$.

To achieve this, we train an autoencoder model 𝒜 consisting of an encoder module ℰ and a decoder module 𝒟. The encoder was responsible for mapping input spectra x into latent space representations z=ℰ(x), and the decoder was responsible for mapping these latent representations into reconstructions of the original input $\hat{x}=𝒟\left(z\right)=𝒟\left[ℰ\left(x\right)\right]=𝒜\left(x\right)$. The model was trained in an unsupervised manner by minimizing the reconstruction error between the input x and the output $\hat{x}$. During this process, the model was guided to learn the endmember components M and their relative abundances A through the introduction of related physical constraints. Below, we provide additional details about the developed architecture and training procedure. For more information about hyperspectral unmixing, the reader is pointed to previous works by Keshava and Mustard (*57*) and Li *et al.* (*58*). For more information about autoencoders, the reader is pointed to the work of Goodfellow *et al.* (*89*).

The encoder ℰ comprised two separate blocks applied sequentially. The first part was a multibranch convolutional block comprising four parallel convolutional layers with kernel sizes of 5, 10, 15, and 20, designed to capture patterns at multiple spectral scales. Each convolutional layer contained 32 filters with rectified linear unit (ReLU) activation, He initialization (*90*), and “same” padding. Batch normalization (*91*) and dropout with a rate of 0.2 (*92*) were applied to each convolutional layer to improve training stability and generalization. The outputs of the four convolutional layers were merged channel-wise through a fully connected layer to yield an output of dimension matching that of the input spectrum. The rationale behind this was to transform intensity values into representations that capture local spectral features (e.g., peak shape, width, and local neighborhood) and thus promote better generalizability. The second part of the encoder was a fully connected dimensionality reduction block, applied to learn patterns between the learned spectral features. This block comprised a series of fully connected layers of sizes 256, 128, 64, and 32 with He initialization and ReLU activation. Batch normalization and dropout (rate of 0.5) were also applied at each fully connected layer. The block was followed by a final fully connected layer [Xavier uniform initialization (*93*)] that reduced the final 32 features to a latent space of size *n*. The number *n* was treated as a hyperparameter that encodes the number of endmembers to extract, with latent representations treated as abundance fractions. To improve interpretation, nonnegativity was enforced in the latent space using a “softly rectified” hyperbolic tangent function $f\left(x\right)=\frac{1}{\gamma }\text{log}\left[1+e^{\gamma *\text{tanh}\left(x\right)}\right]$, with γ = 10, as we previously reported (*55*). This ensured that latent values were constrained to the range (0, 1) in accordance with the abundance nonnegativity constraint but does not enforce abundances to sum to one (abundance sum-to-one constraint).

The decoder 𝒟 consisted of a single fully connected layer (Xavier uniform initialization) mapping the *n*-dimensional latent space representations back to the original spectral dimension *b*. In this layer, we used a linear activation and no bias term. Under this setup, the decoder mimics linear unmixing, where endmember signatures are encoded in the learned weight matrix of the decoder layer (*55*). A nonnegative kernel constraint was used to enforce the nonnegativity of endmember signatures using weight clipping during training. To accelerate model training, the weight matrix in the linear decoder layer was initialized before training using endmember signatures derived with VCA (*66*).

Autoencoder models were trained for 5 epochs using the Adam optimizer (*94*) with a batch size of 32. For our time point study, training epochs were reduced to 2. The learning rate was set to 0.001 for our two cryosection scans and subcellular day-20 scan, or 0.003 for all other data. An exponential learning rate decay was used to ensure stable convergence, applying a factor of 0.9 to the learning rate after each epoch. The training loss ℒ was defined as a weighted sum of the mean squared error (MSE) and spectral angle divergence (SAD) between input spectra x and output reconstructions $\hat{x}$

$$ℒ\left(x,\hat{x}\right)=\text{SAD}\left(x,\hat{x}\right)+\lambda \;\text{MSE}\left(x,\hat{x}\right)$$ (1)

where

$$\text{MSE}\left(x,\hat{x}\right)=\frac{1}{b}{\left\|x-\hat{x}\right\|}_{2}$$ (2)

with *b* denoting the dimension of x (i.e., the number of spectral bands), and

$$\text{SAD}\left(x,\hat{x}\right)=\text{arccos}\left(\frac{x\cdot \hat{x}}{∥\;x\;{∥}_{2}\;∥\;\hat{x}\;{∥}_{2}}\right)$$ (3)

The weighting factor λ was set to 10,000.

The number of endmembers to derive was estimated individually for each scan/dataset of interest using PCA. Specifically, we computed the cumulative explained variance as a function of the number of principal components and manually selected the number of components at the point where the increase in explained variance visually plateaued.

The choice of endmember number is a key hyperparameter in blind unmixing as it directly affects the resulting abundance maps: Underestimation can lead to the mixing of distinct spectral components, whereas overestimation can split a single component across multiple endmembers. While our PCA-based criterion still involves a degree of manual judgement and can be sensitive to factors such as signal-to-noise ratio, background contributions, preprocessing choices, and the intrinsic heterogeneity of biological tissue, it provides a transparent, data-driven diagnostic that is deterministic across runs. Our sensitivity analysis in fig. S13 showed that our pipeline is robust to reasonable variation around the chosen value.

Autoencoder model implementation, training, and evaluation steps were carried out using TensorFlow (*95*). Analysis with baseline chemometric methods was performed using RamanSPy (*56*).

### Time point analysis

Quantitative analysis is based on Raman imaging data from three batches of cerebral organoids, cultured in independent experiments using stem cells at different passage numbers. For our quantification analysis, manual background segmentation was performed using the derived abundance reconstructions as a reference. The obtained binary masks were used to (i) discard background pixels by setting abundance values in the background area to zero for all channels and (ii) estimate the relative tissue coverage in each scan. Scans with an organoid coverage below 30% were not used for the subsequent quantification to avoid potential biasing toward components in the outer tissue layers. In total, 6 scans of the available 72 were discarded (fig. S19B). Last, the mean abundance of endmembers was calculated for each scan and normalized by the organoid area. Representative abundance images were produced without background removal, after scaling the data by a factor of 2 in *z* (organoid depth) to achieve an equal effective pixel size of 5 μm in *x-y* and *z*, and thus provide a more accurate depiction of the organoid morphology and dimensions.

Automated segmentation using Otsu thresholding was also tested but yielded inconsistent masks with substantial speckling, fragmentation and misclassified pixels (fig. S22), which could not be adequately corrected through morphological operations such as erosion and dilation. We acknowledge, however, that manual masking is time-intensive and potentially subject to operator bias. Transitioning to high-throughput workflows will therefore necessitate more robust automated approaches, such as advanced abundance thresholding with refined morphological clean-up or custom deep learning–based segmentation models.

## Supplementary Materials

### This PDF file includes:

Tables S1 and S2 Figs. S1 to S24 References
