# Supplementary material for: Label-free biochemical imaging and time point analysis of neural organoids via deep learning–enhanced Raman microspectroscopy
Source: Sci Adv. 2026 Aug 12;12(33):eaec5080. doi: 10.1126/sciadv.aec5080 (PMC13464642; doi:10.1126/sciadv.aec5080)
Supplement: Supplementary file 1 [file sciadv.aec5080_sm.pdf]

Science Advances
AAAS

Supplementary Materials for

Label-free biochemical imaging and time point analysis of neural organoids via deep learning–enhanced Raman microspectroscopy

Dimitar Georgiev *et al.*

Corresponding author: Mauricio Barahona, m.barahona@imperial.ac.uk;
Molly M. Stevens, molly.stevens@dpag.ox.ac.uk

Sci. Adv. **12**, eaec5080 (2026)
DOI: 10.1126/sciadv.aec5080

This PDF file includes:

Tables S1 and S2
Figs. S1 to S24
References

Table S1: Primary antibodies. Extended information about the primary antibodies used for immunofluorescence staining. 'Cat. no.' stands for 'Catalogue number'.

Figure panel(s)	Antigen	Species	Producer	Cat. no.	RRID	Dilution
Figure 1C	SOX2	Rabbit	Abcam	ab97959	AB_2341193	1:600
	PKCζ	Mouse	Santa Cruz Biotechnology	sc-17781	AB_628148	1:200
Figures 2C, S4B and S6C	SOX2	Goat	R&D Systems	AF2018	AB_355110	1:100
	TUJ1	Mouse	BioLegend	801201	AB_2313773	1:100
Figures 2D and S5B	SOX2	Goat	R&D Systems	AF2018	AB_355110	1:100
	TUJ1	Mouse	BioLegend	801201	AB_2313773	1:100
Figure 4A	SOX2	Rabbit	Abcam	ab97959	AB_2341193	1:600
	PKCζ	Mouse	Santa Cruz Biotechnology	sc-17781	AB_628148	1:200
	MAP2	Chicken	Abcam	ab5392	AB_2138153	1:500

Table S2: Secondary antibodies. Extended information about the secondary antibodies used for immunofluorescence staining. 'Cat. no.' stands for 'Catalogue number'.

Figure panel(s)	Species	Anti-	Producer	Cat. no.	RRID	Dilution
Figure 1C	Goat	Rabbit	Invitrogen	A-11008	AB_143165	1:500
	Donkey	Mouse	Invitrogen	A-31571	AB_162542	1:500
Figures 2C, S4B and S6C	Donkey	Goat	Invitrogen	A-21432	AB_141788	1:500
	Donkey	Mouse	Invitrogen	A-31571	AB_162542	1:500
Figures 2D and S5B	Donkey	Goat	Invitrogen	A-21432	AB_141788	1:500
	Donkey	Mouse	Invitrogen	A-31571	AB_162542	1:500
Figure 4A	Goat	Rabbit	Invitrogen	A-11008	AB_143165	1:500
	Donkey	Mouse	Invitrogen	A-31571	AB_162542	1:500
	Goat	Chicken	Invitrogen	A-21437	AB_2535858	1:500

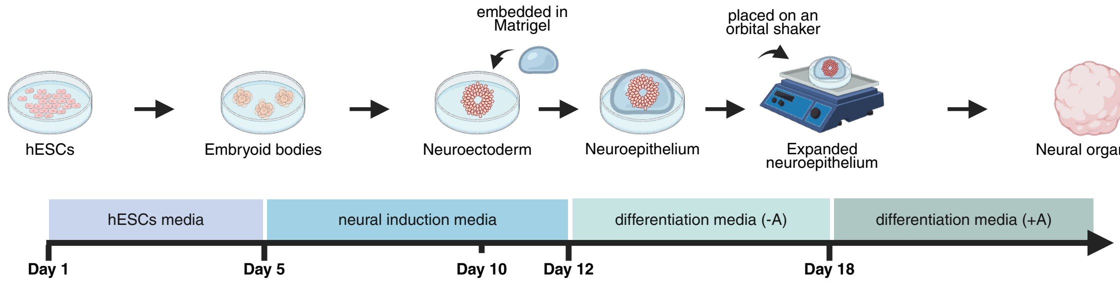

Figure S1: Schematic diagram of organoid culture. Neural organoids were grown using a protocol for unguided differentiation based on previous work from Lancaster et al. (7). Created in BioRender. Georgiev, D. (2026). https://BioRender.com/1y0chm0.

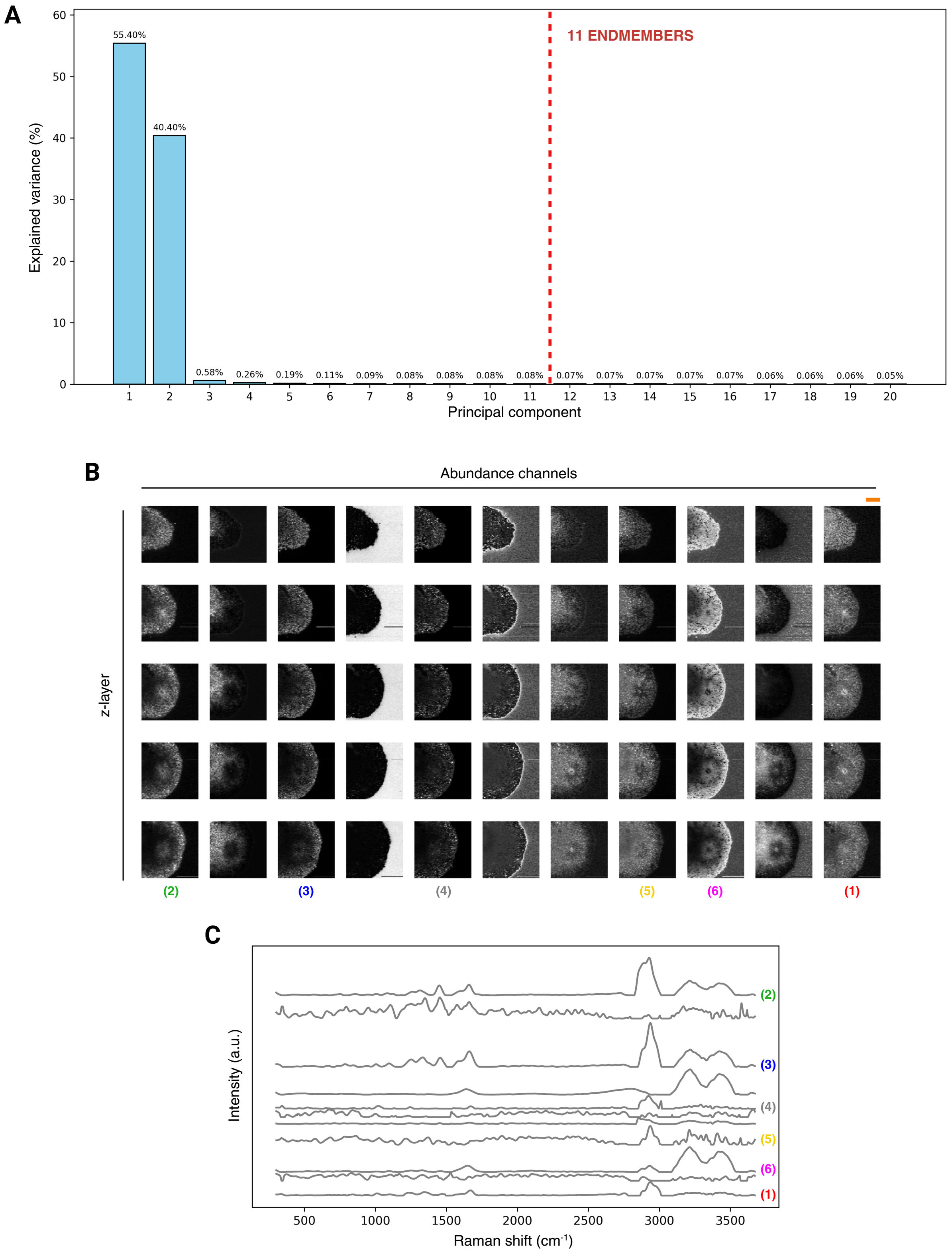

Figure S2: Extended unmixing results for the low-resolution volumetric Raman imaging scan of an intact day-20 neural organoid (as shown in Figures 1D–I in the main text). A, Principal component analysis of preprocessed RS data. The number of endmember components to extract was set to 11. **B,** Derived abundance images (normalised for visualisation purposes). Scale bar: 100 µm (top right, marked in orange). **C,** Corresponding endmembers. The endmember and abundance numbers indicate the components presented in the main text. Raman data were acquired using the following parameters: pixel size = 5 µm in *x*-*y* and 10 µm in *z*, integration time = 0.5 second, laser power ≈ 32 mW, objective = 63×, NA 1. Created in BioRender. Georgiev, D. (2026). https://BioRender.com/p7lhiuz.

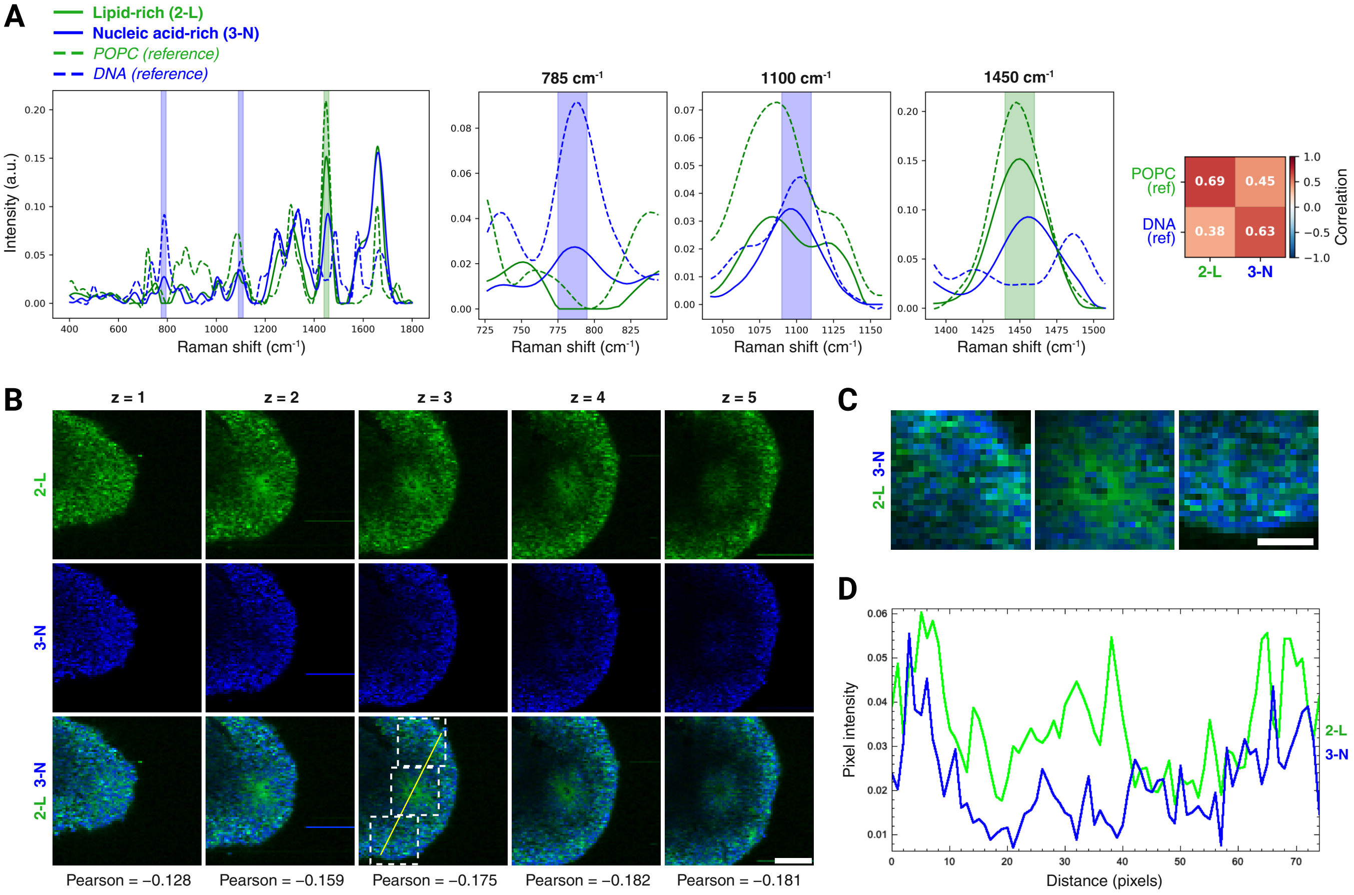

Figure S3: Spectral and spatial analysis of derived endmembers related to nucleic acids and lipids (as shown in Figures 1D–I in the main text). A, Lipid-rich (2-L, green) and nucleic acid-rich (3-N, blue) endmember spectra, overlaid with reference spectra of POPC (phospholipid) and DNA from LaLone et al. (53) (dashed). Shaded windows mark canonical nucleic acid ($\sim$785 cm^{-1} and $\sim$1100 cm^{-1}) and lipid ($\sim$1450 cm^{-1}) bands, with expanded views shown for each band. Heatmap shows the Pearson correlation coefficient between derived endmembers and reference spectra (also presented in Figure 1I). **B,** Abundance maps of 2-L and 3-N across the five z-planes (10 µm step in z). Pearson indicates the pixelwise Pearson correlation coefficient between the abundance images of 2-L and 3-N for each position in z. Scale bar: 100 µm. **C,** Three zoomed-in regions of interest (marked in **B** with white dashed boxes, $z = 3$). Scale bar: 50 µm. **D,** Line profiles along the transect in **B** (marked as a yellow line, $z = 3$), showing non-identical spatial variation (1 pixel = 5 µm). Raman data were acquired using the following parameters: pixel size = 5 µm in x-y and 10 µm in z, integration time = 0.5 second, laser power $\approx$ 32 mW, objective = 63×, NA 1. Created in BioRender. Georgiev, D. (2026). https://BioRender.com/0c44csh.

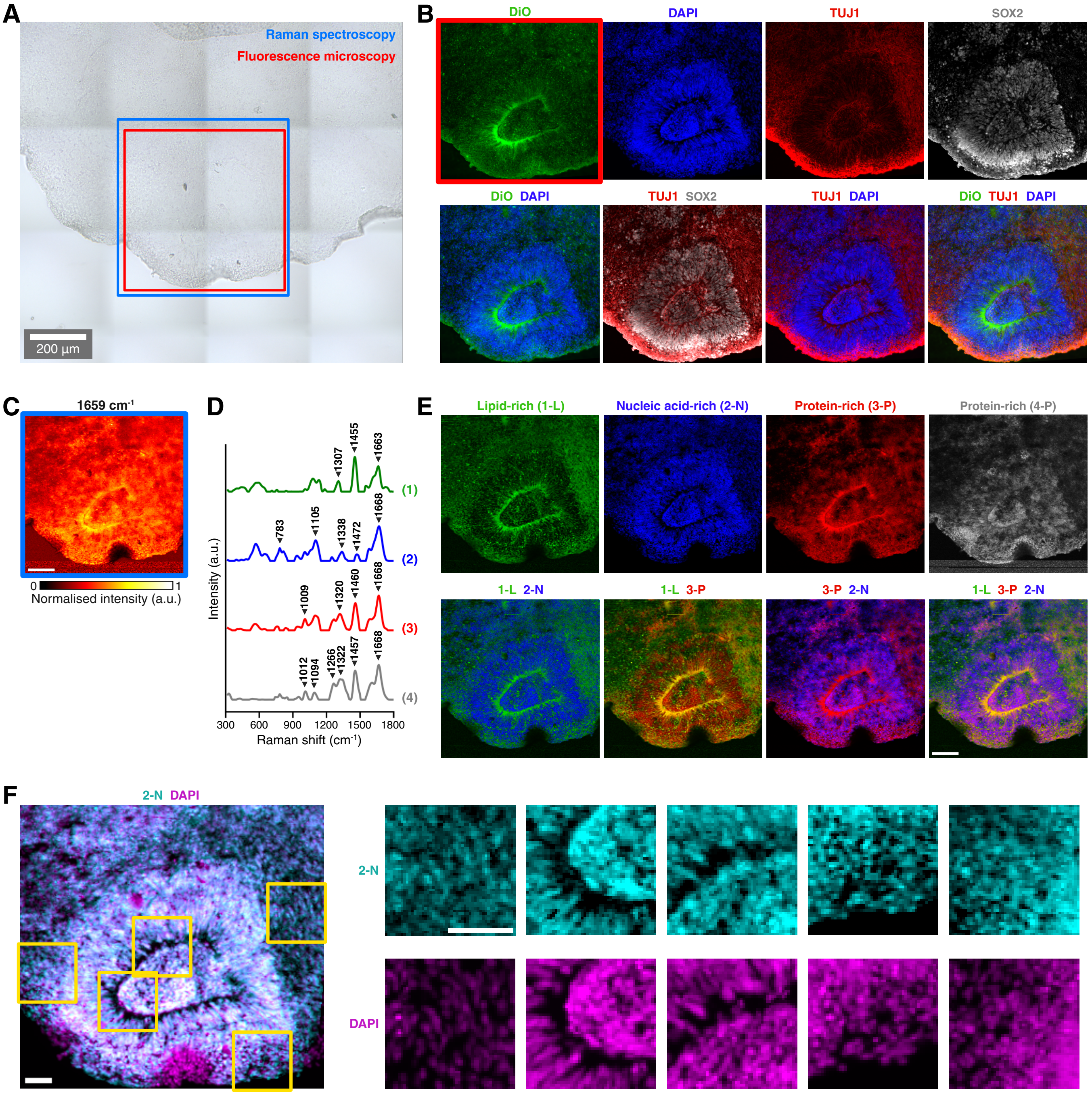

Figure S4: See next page for caption.

Figure S4: Extended results for the Raman imaging scan of the first day-30 neural organoid cryosection (as shown in Figures 2B and 2C in the main text). **A,** Brightfield image of a sectioned neural organoid tissue region imaged using Raman spectroscopy (blue) and fluorescence microscopy (red). Scale bar: 200 µm. The brightfield image is also presented in Figure 2B (*left*). **B,** Fluorescence microscopy images with markers for nuclei (DAPI), membranes (DiO), neural progenitors (SOX2) and neurons (TUJ1). DiO, DAPI and DiO + DAPI staining images were selected for presentation in Figure 2C. **C,** Raman intensity profile at the 1659 cm^{-1} band (linked to proteins and lipids). Scale bar: 100 µm. **D–E,** Results of hyperspectral unmixing analysis. **D,** Derived tissue-specific endmember signatures. Endmembers scaled for visualisation purposes (maximum intensity set to 1). **E,** Corresponding abundance maps. Scale bar: 100 µm. Endmember 1 (green) presented a lipid-rich spectral signature with distinct peaks at 1307 cm^{-1}, 1455 cm^{-1} and 1663 cm^{-1}. Its abundance was consistent with membrane structures, including cellular membranes and the apical domain of the visible neural rosette. The signal of this endmember showed a high degree of visual overlap with DiO and TUJ1. Endmember 2 (blue) displayed a nucleic acid-rich profile with a prominent band at 783 cm^{-1}, alongside vibrations linked to proteins (1338 cm^{-1}, 1472 cm^{-1} and 1668 cm^{-1}). Strong glass signal was also observed around 570 cm^{-1} and 1100 cm^{-1}. The abundance map revealed well-defined, spherical compartments, closely resembling cell nuclei, with increased density within the present neural rosette structure. Endmembers 3 (red) and 4 (grey) shared similar spectral signatures, dominated by protein-associated peaks around $\sim$1010 cm^{-1}, $\sim$1100 cm^{-1}, $\sim$1265 cm^{-1}, $\sim$1320 cm^{-1}, $\sim$1460 cm^{-1} and $\sim$1670 cm^{-1}. Both endmembers were generally broadly distributed across the sample. Endmember 3 showed increased signal at the apical domain of the present rosette, potentially suggesting structural proteins. The abundance of endmember 4 was increased within the rosette structure. 1-L, 2-N and 1-L + 2-N images were selected for presentation in Figure 2C. **F,** Overlaid images of DAPI and endmember associated with nucleic acids (2-N). Scale bars: 25 µm. Raman data were acquired using the following parameters: pixel size = 2 µm in *x*-*y*, integration time = 0.5 second, laser power $\approx$ 48 mW, objective = 20$\times$, NA 0.5. Created in BioRender. Georgiev, D. (2026). https://BioRender.com/g0nv7qq.

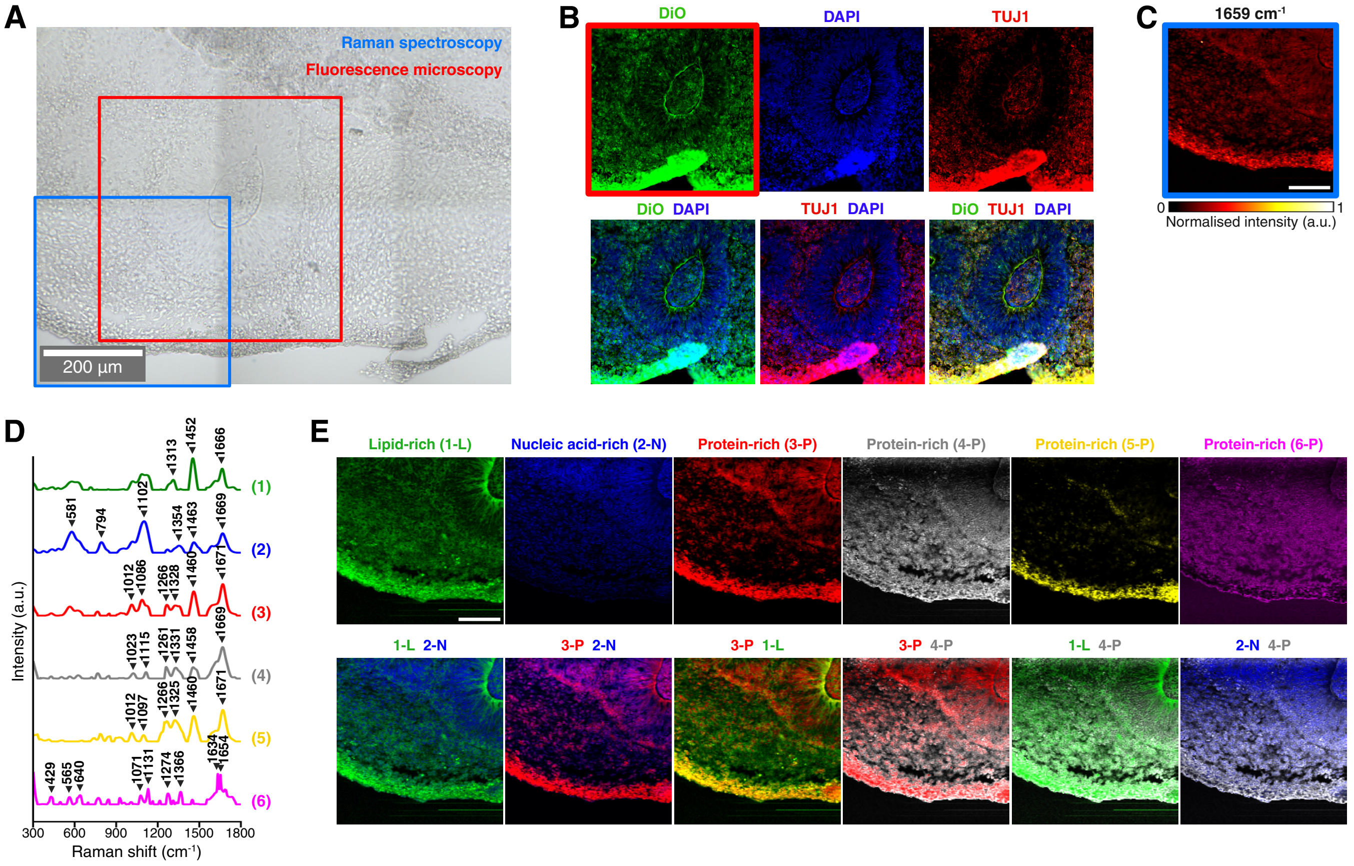

Figure S5: Extended results for the Raman imaging scan of the second day-30 neural organoid cryosection (as shown in Figures 2B and 2D in the main text). A, Brightfield image of a sectioned neural organoid tissue imaged using Raman spectroscopy (blue) and fluorescence microscopy (red). Scale bar: 200 µm. The brightfield image is also presented in Figure 2B (*right*). **B,** Fluorescence microscopy images with markers for nuclei (DAPI), membranes (DiO) and neurons (TUJ1). Due to partial tissue detachment during the staining process, a localised increase in fluorescence intensity was observed at the tissue edge. This peripheral region was identified as an artefact and was therefore excluded from subsequent analyses. Selected area imaged with both fluorescence microscopy and RS is presented in Figure 2D – channels DiO, DAPI and DiO + DAPI. **C,** Raman intensity profile at the 1659 cm^{-1} band (linked to proteins and lipids). Scale bar: 100 µm. **D–E,** Results of hyperspectral unmixing analysis. **D,** Derived tissue-specific endmember signatures. Endmembers scaled for visualisation purposes (maximum intensity set to 1). **E,** Corresponding abundance maps. Scale bar: 100 µm. Endmember 1 (green) exhibited a lipid-rich profile with prominent peaks at 1313 cm^{-1}, 1452 cm^{-1} and 1666 cm^{-1}. The corresponding abundance map presented morphological architecture consistent with membrane-like structures, including individual cellular membranes and the apical domain of the present neural rosette. Endmember 2 (blue) displayed a strong nucleic acid band at 794 cm^{-1}, alongside several vibrations linked to protein species (1354 cm^{-1}, 1463 cm^{-1} and 1669 cm^{-1}), as well as bands attributed to the glass slide at 581 cm^{-1} and 1102 cm^{-1}. The abundance map revealed well-defined, spherical compartments, closely resembling cell nuclei. Endmembers 3 (red), 4 (grey) and 5 (yellow) exhibited similar spectral signatures, dominated by protein-associated peaks around ∼1015 cm^{-1}, ∼1100 cm^{-1}, ∼1265 cm^{-1}, ∼1330 cm^{-1}, ∼1460 cm^{-1} and ∼1670 cm^{-1}. Endmember 4 showed a broad spatial distribution, likely corresponding to cytoplasmic or general background tissue signal. Endmembers 3 and 5 showed a more pronounced 1460 cm^{-1} band and an increased signal near the edge of the tissue section, likely due to tissue curling, as well as the basal domains of the present neural rosettes. Endmember 6 (magenta) presented a noisier signature, with prominent protein-associated peaks at 1131 cm^{-1} (C–N stretching), 1274 cm^{-1} and 1366 cm^{-1} (tryptophan). Its spatial distribution showed substantial overlap with that of Endmember 4. Selected area imaged with both fluorescence microscopy and RS is presented in Figure 2D – channels 1-L, 2-N and 1-L + 2-N. Raman data were acquired using the following parameters: pixel size = 2 µm in *x-y*, integration time = 0.5 second, laser power ≈ 48 mW, objective = 20×, NA 0.5. Created in BioRender. Georgiev, D. (2026). https://BioRender.com/636f0fe.

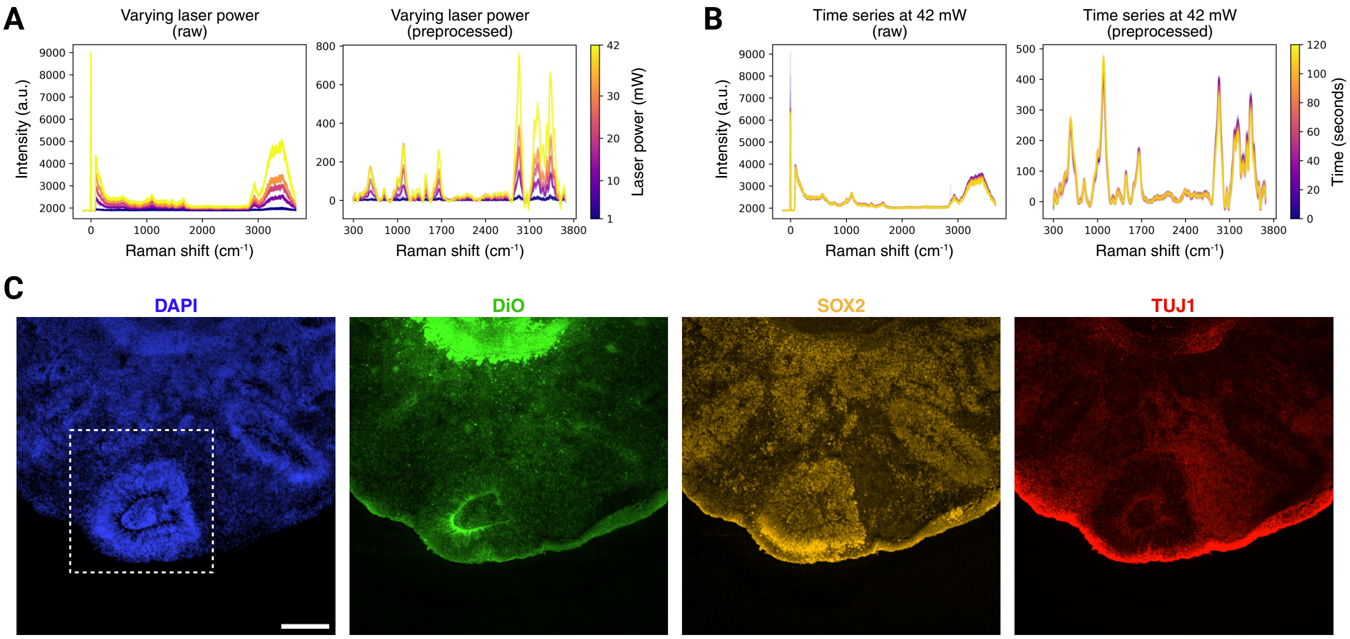

Figure S6: Assessment of possible photodamage effects. A, Raman spectra from a fixed location in a neural organoid cryosection acquired at varying laser power setpoints in the WITec control software, namely 1 mW ($n = 6$), 10 mW ($n = 3$), 20 mW ($n = 3$), 30 mW ($n = 3$) and 42 mW ($n = 3$). **B,** Time series Raman acquisition from a fixed location with a laser power setpoint of 42 mW (corresponding to $\sim$48 mW measured at the sample with a power meter), showing stable spectra under prolonged illumination (120 seconds in total; one spectrum per second). Raman data were acquired using the following parameters: integration time = 1 second, objective = 20×, NA 0.5. **C,** Full fluorescence microscopy images of the first neural organoid cryosection (selected areas are shown in Figures 2C and S4B). Fluorescence channels: DAPI (nuclei), DiO (membranes), SOX2 (neural progenitors) and TUJ1 (neurons). The area previously scanned by Raman imaging is indicated by a white dashed box. No visible effects on fluorescence signal, morphology or staining were observed. Scale bar: 200 µm. Created in BioRender. Georgiev, D. (2026). https://BioRender.com/clsx46x.

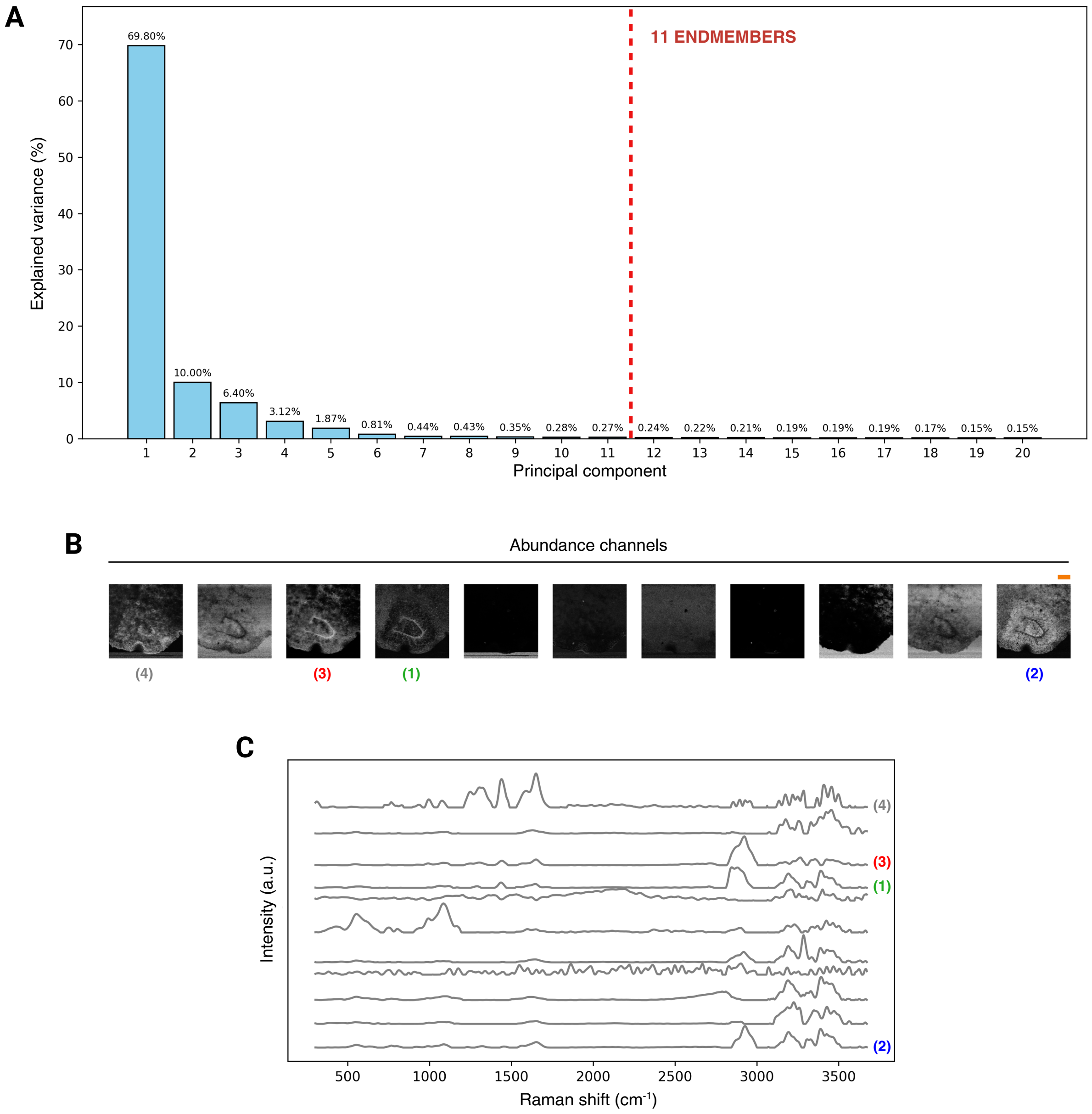

Figure S7: Extended unmixing results for the Raman imaging scan of the first day-30 neural organoid cryosection (as shown in Figures 2B and 2C in the main text). A, Principal component analysis of preprocessed RS data. The number of endmember components to extract was set to 11. **B,** Derived abundance images (normalised for visualisation purposes). Scale bar: 100 µm (top right, marked in orange). **C,** Corresponding endmembers. The endmember and abundance numbers indicate the components presented in the main text. Raman data were acquired using the following parameters: pixel size = 2 µm in *x*-*y*, integration time = 0.5 second, laser power ≈ 48 mW, objective = 20×, NA 0.5. Created in BioRender. Georgiev, D. (2026). https://BioRender.com/4d79l8i.

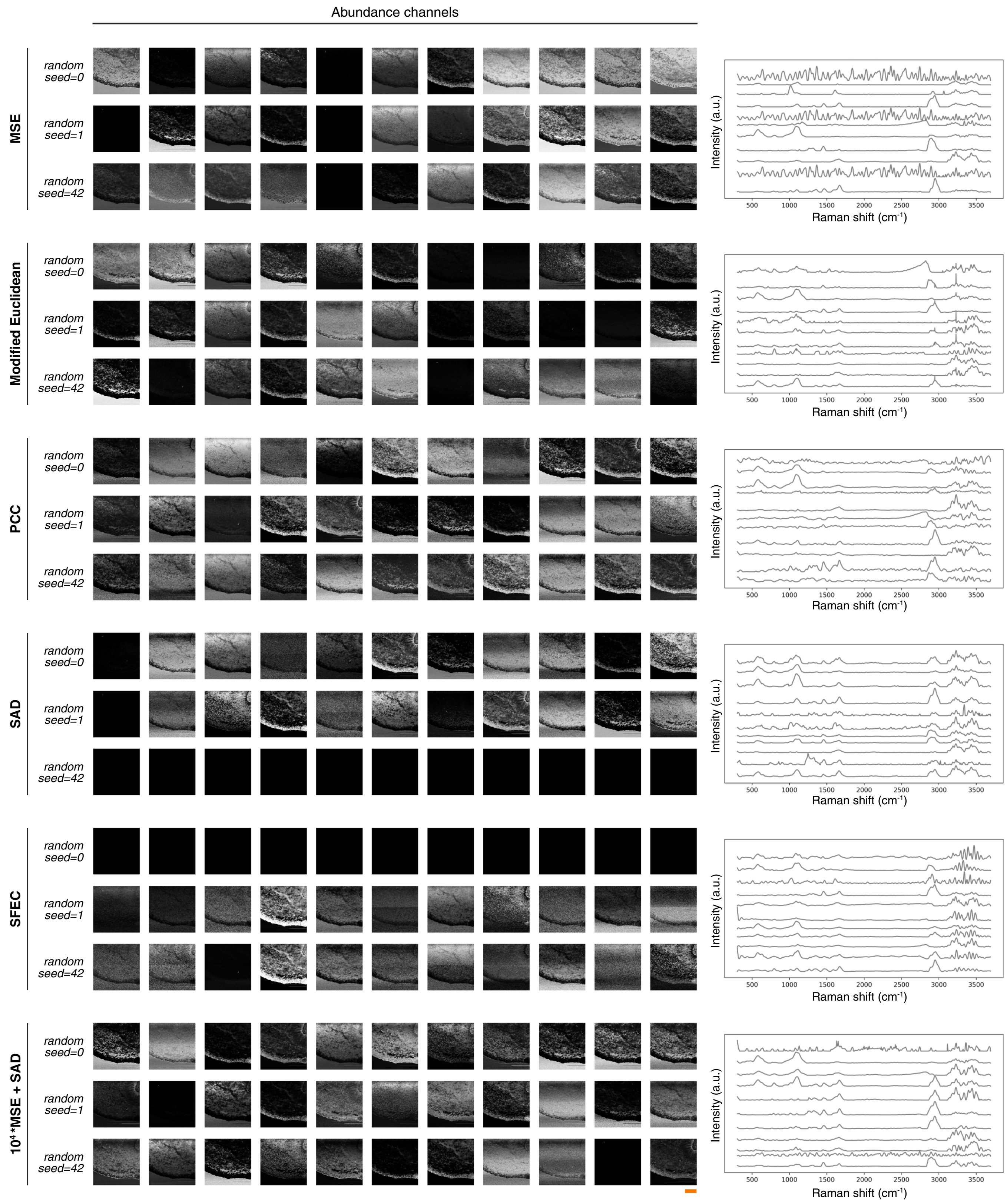

Figure S8: See next page for caption.

Figure S8: Effect of training loss on autoencoder performance on the Raman imaging scan of the second day-30 neural organoid cryosection (as shown in Figures 2B and 2D in the main text). Losses tested include: mean squared error (MSE), modified Euclidean (96), Pearson's correlation coefficient (PCC), spectral angle divergence (SAD) (59), squared first-difference Euclidean cosine (SFEC) (97), and our custom loss based on a combination of MSE and SAD. Abundance images were normalised for visualisation purposes. Endmember signatures displayed on the right correspond to the analyses performed with a random seed set to 42. Scale bar: 100 µm (bottom right, marked in orange). Raman data were acquired using the following parameters: pixel size = 2 µm in *x*-*y*, integration time = 0.5 second, laser power ≈ 48 mW, objective = 20×, NA 0.5. Created in BioRender. Georgiev, D. (2026). https://BioRender.com/6p5c1jt.

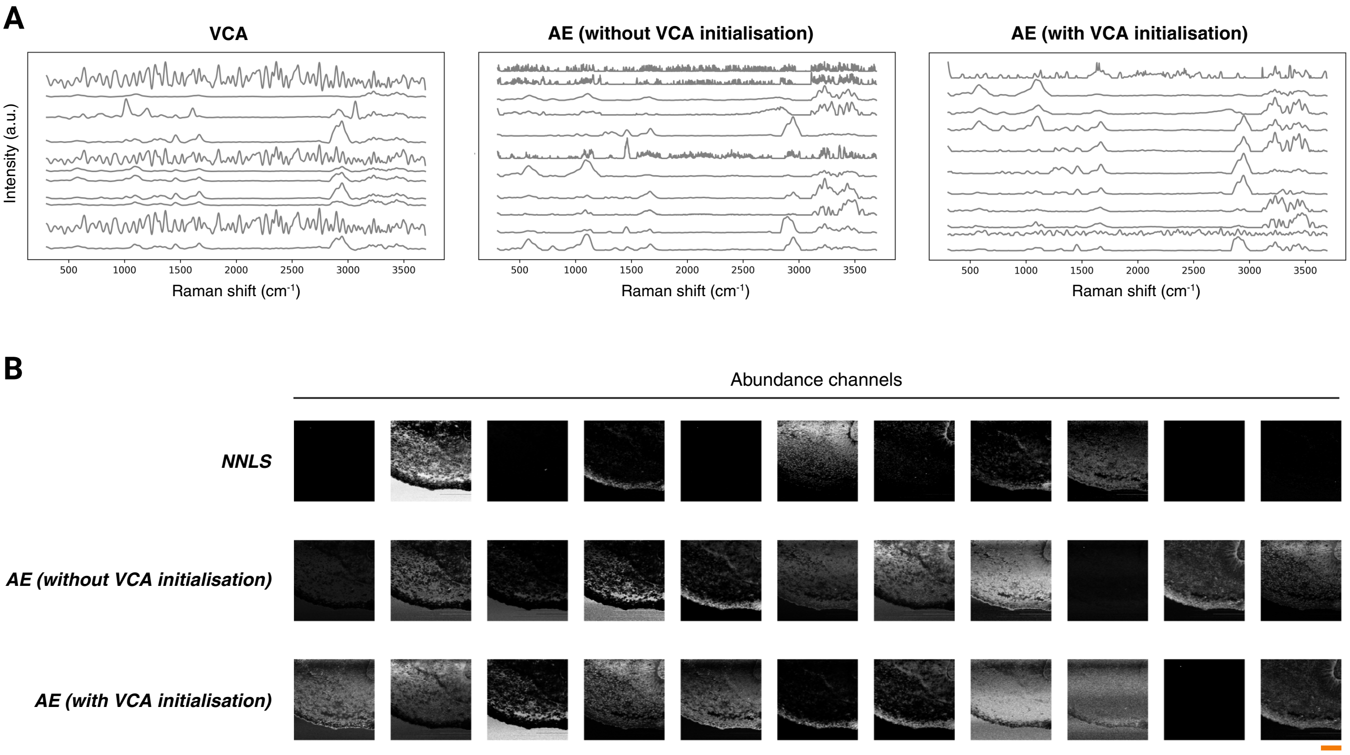

Figure S9: Effect of endmember initialisation on autoencoder performance on the Raman imaging scan of the second day-30 neural organoid cryosection (as shown in Figures 2B and 2D in the main text). **A,** Derived endmembers with (i) vertex component analysis (VCA), (ii) our autoencoder model without VCA initialisation, and (iii) our autoencoder model with VCA initialisation. **B,** Derived abundances with (i) non-negative least squares (NNLS) performed using the VCA endmembers displayed in **A**, (ii) our autoencoder model without VCA initialisation, and (iii) our autoencoder model with VCA initialisation. Abundance images were normalised for visualisation purposes. Scale bar: 100 µm (bottom right, marked in orange). Raman data were acquired using the following parameters: pixel size = 2 µm in *x*-*y*, integration time = 0.5 second, laser power ≈ 48 mW, objective = 20×, NA 0.5. Created in BioRender. Georgiev, D. (2026). https://BioRender.com/d8ac23y.

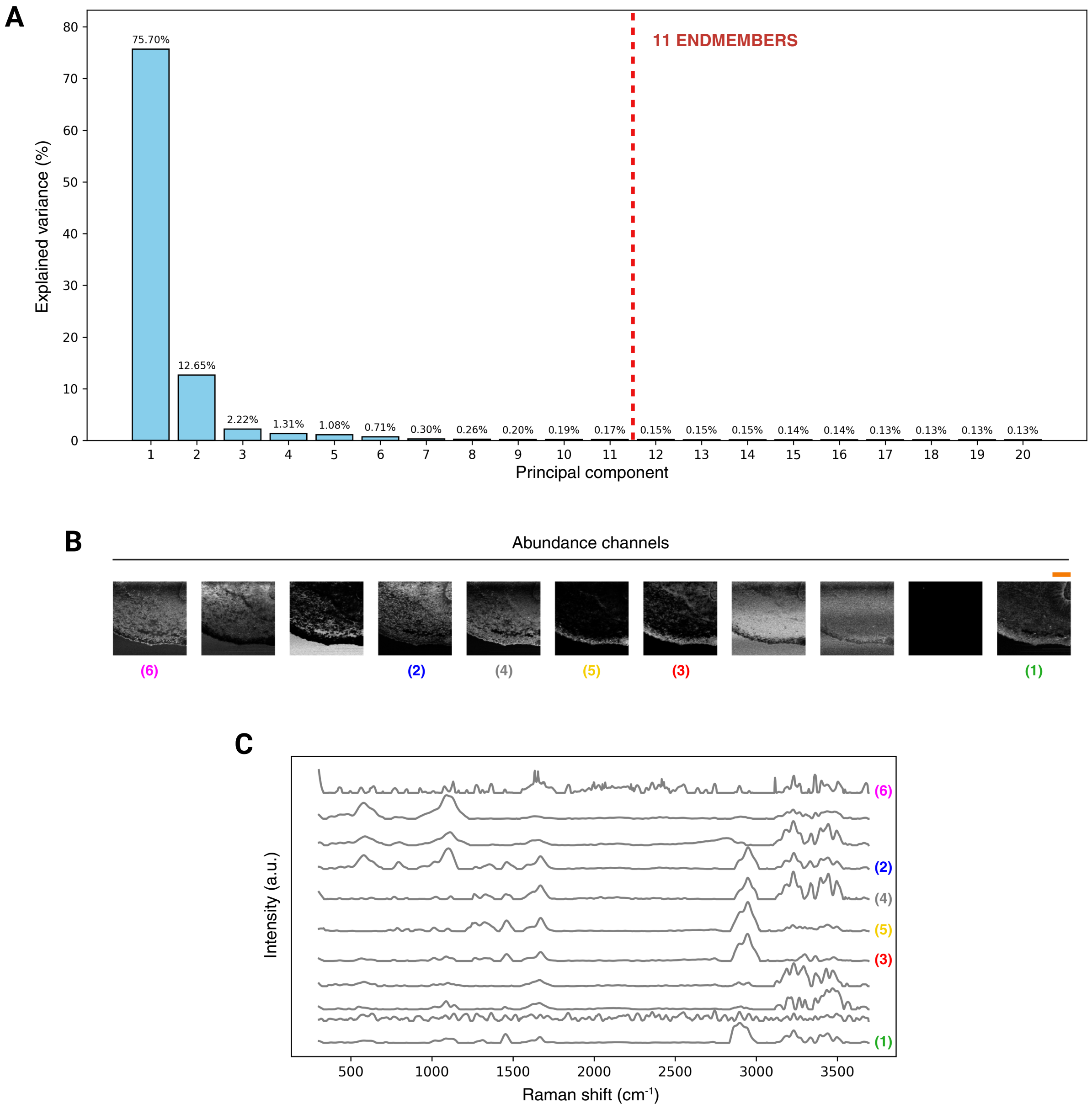

Figure S10: Extended unmixing results for the Raman imaging scan of the second day-30 neural organoid cryosection (as shown in Figures 2B and 2D in the main text). A, Principal component analysis of preprocessed RS data. The number of endmember components to extract was set to 11. **B,** Derived abundance images (normalised for visualisation purposes). Scale bar: 100 µm (top right, marked in orange). **C,** Corresponding endmembers. The endmember and abundance numbers indicate the components presented in the main text. Raman data were acquired using the following parameters: pixel size = 2 µm in *x*-*y*, integration time = 0.5 second, laser power ≈ 48 mW, objective = 20×, NA 0.5. Created in BioRender. Georgiev, D. (2026). https://BioRender.com/mnimvuo.

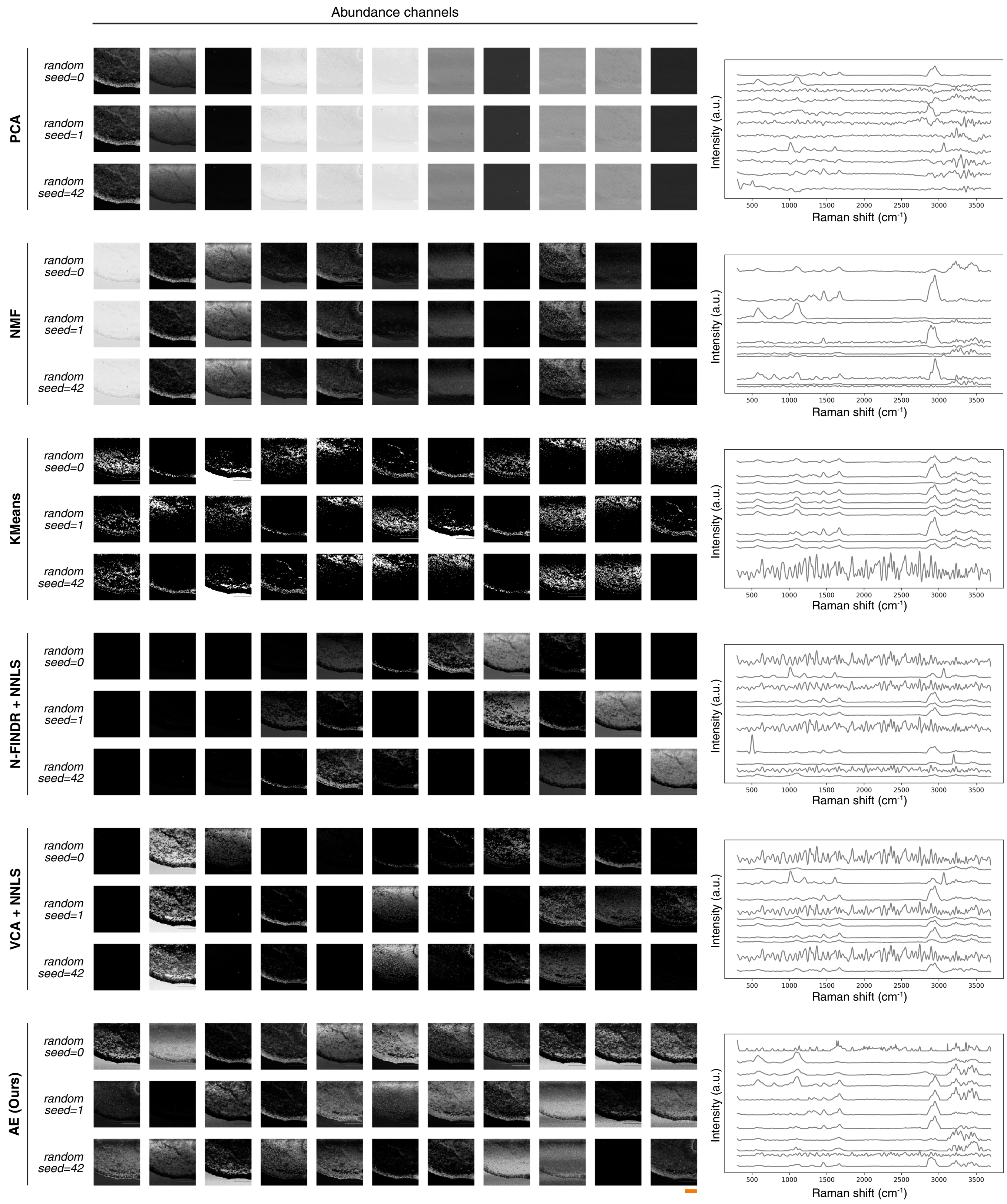

Figure S11: See next page for caption.

Figure S11: Comparative analysis of different chemometric methods on the Raman imaging scan of the second day-30 neural organoid cryosection (as shown in Figures 2B and 2D in the main text). Methods include: principal component analysis (PCA), non-negative matrix factorisation (NMF), k-means clustering, N-FINDR + non-negative least squares (NNLS), vertex component analysis (VCA) + NNLS, our autoencoder (AE) pipeline. Abundance images were normalised for visualisation purposes. Endmember signatures displayed on the right correspond to the analyses performed with a random seed set to 42. PCA yielded a single tissue-specific component – its first principal component – that effectively captured the overall tissue architecture. The remaining components displayed convoluted abundances with high uniform background signals and negligible biochemical specificity. In contrast, NMF identified three distinct endmembers, resembling major molecular species such as protein-rich, lipid-rich and nucleic acid-rich components. However, it was unable to resolve finer biochemical features beyond these primary categories. K-means clustering showed limited biochemical interpretability, segmenting the data into different tissue regions, possibly due to intensity variation instead of meaningful compositional components. More specialised unmixing techniques like N-FINDR+NNLS exhibited serious susceptibility to artefacts, producing multiple abundance maps with isolated "noisy" pixels. Beyond one general tissue-level component, N-FINDR+NNLS failed to resolve distinct cellular structures such as nuclei or membranes. VCA+NNLS demonstrated improved performance relative to N-FINDR+NNLS, producing five distinct abundance maps that captured features such as nuclei, the apical domain of the imaged neural rosette and potentially cytoplasmic regions. Nonetheless, some artefacts and shading inconsistencies persisted – for instance, within the nuclear component (channel 6), which displayed a strong signal in the upper field that markedly diminished toward the bottom. Scale bar: 100 µm (bottom right, marked in orange). Raman data were acquired using the following parameters: pixel size = 2 µm in *x*-*y*, integration time = 0.5 second, laser power ≈ 48 mW, objective = 20×, NA 0.5. Created in BioRender. Georgiev, D. (2026). https://BioRender.com/bgrihln.

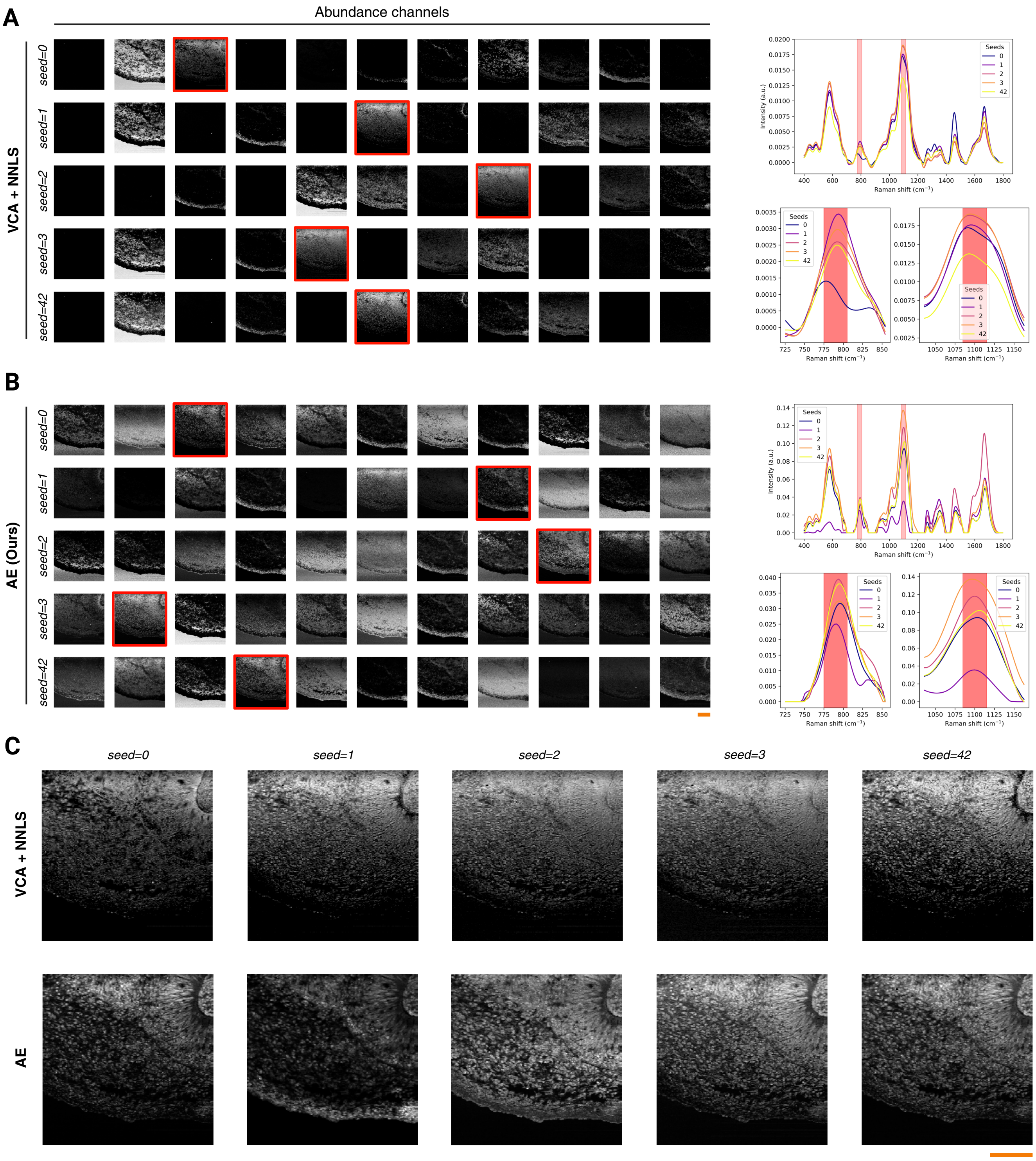

Figure S12: Unmixing variability across random seeds. Spectral unmixing of the second cryosection scan (as shown in Figures 2B and 2D in the main text) across five random seeds using VCA + NNLS (**A**) and our autoencoder-based pipeline (**B**), with all other parameters held constant. Red boxes highlight the nucleic acid-rich component derived in each run. Panels on the right show the corresponding nucleic acid-rich endmembers across seeds, highlighting characteristic nucleic acid-associated Raman bands ($\sim$785 cm^{-1} and $\sim$1100 cm^{-1}; shaded in red). **C,** Abundance maps of nucleic acid-associated endmembers. Scale bars: 100 µm (marked in orange). Raman data were acquired using the following parameters: pixel size = 2 µm in *x*-*y*, integration time = 0.5 second, laser power ≈ 48 mW, objective = 20×, NA 0.5. Created in BioRender. Georgiev, D. (2026). https://BioRender.com/6h85440.

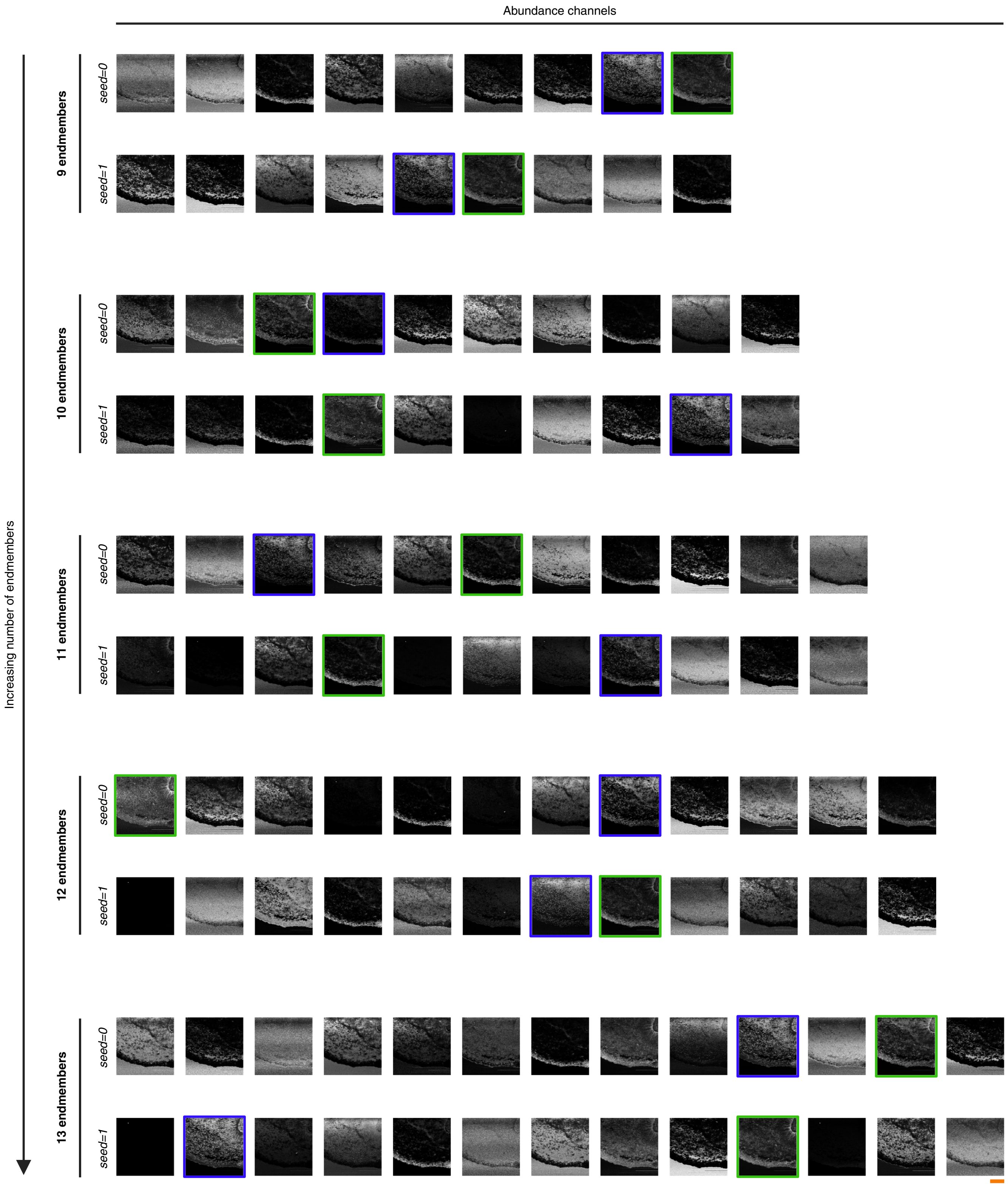

Figure S13: Robustness to the number of extracted endmembers. Unmixing results for the second cryosection scan (as shown in Figures 2B and 2D in the main text) using 9–13 endmembers (original setting: 11), with two random seeds per setting and all other parameters held constant. Nucleic acid-rich and lipid-rich components highlighted in blue and green, respectively. Scale bar: 100 µm (bottom right, marked in orange). Raman data were acquired using the following parameters: pixel size = 2 µm in *x*-*y*, integration time = 0.5 second, laser power ≈ 48 mW, objective = 20×, NA 0.5. Created in BioRender. Georgiev, D. (2026). https://BioRender.com/0d4blbd.

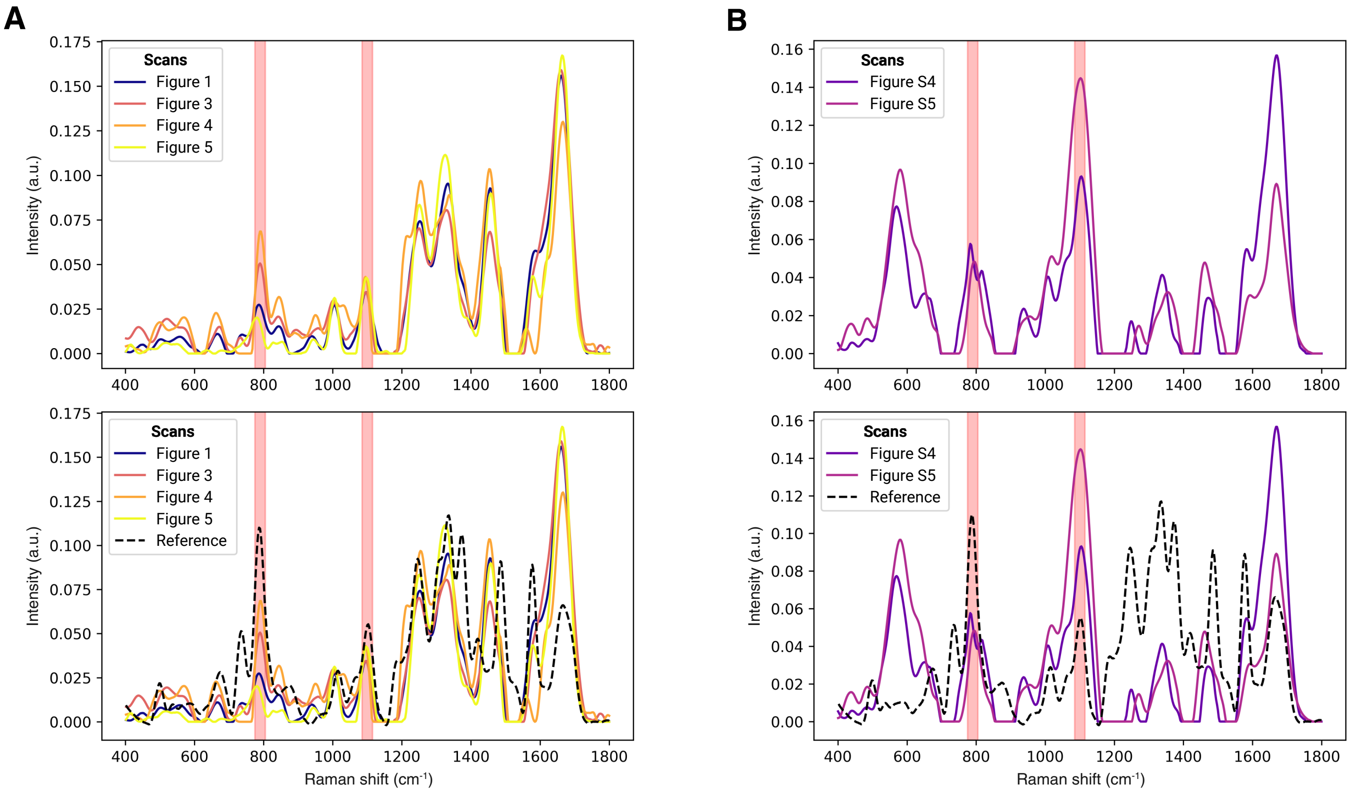

Figure S14: Consistency of nucleic acid-rich endmembers across scans/datasets. Derived nucleic acid-rich endmembers across intact samples (**A**) and cryosectioned samples (**B**). Bottom panels show the endmembers alongside a DNA reference spectrum by LaLone et al. (53). Shaded regions highlight the two canonical bands used for nucleic acid assignment ($\sim$785 cm^{-1} and $\sim$1100 cm^{-1}). Mean pairwise Pearson correlation between endmember spectra was $R = 0.92 \pm 0.04$ for intact datasets (six pairwise comparisons among four datasets) and $R = 0.75$ for cryosection datasets (single pairwise comparison among two datasets). Raman data from intact samples (**A**) were acquired using the following parameters: pixel size = 1 µm–5 µm in *x*-*y*, integration time = 0.5 second, laser power ≈ 32 mW, objective = 63×, NA 1. Raman data from cryosectioned samples (**B**) were acquired using the following parameters: pixel size = 2 µm in *x*-*y*, integration time = 0.5 second, laser power ≈ 48 mW, objective = 20×, NA 0.5. Created in BioRender. Georgiev, D. (2026). https://BioRender.com/fi22c0t.

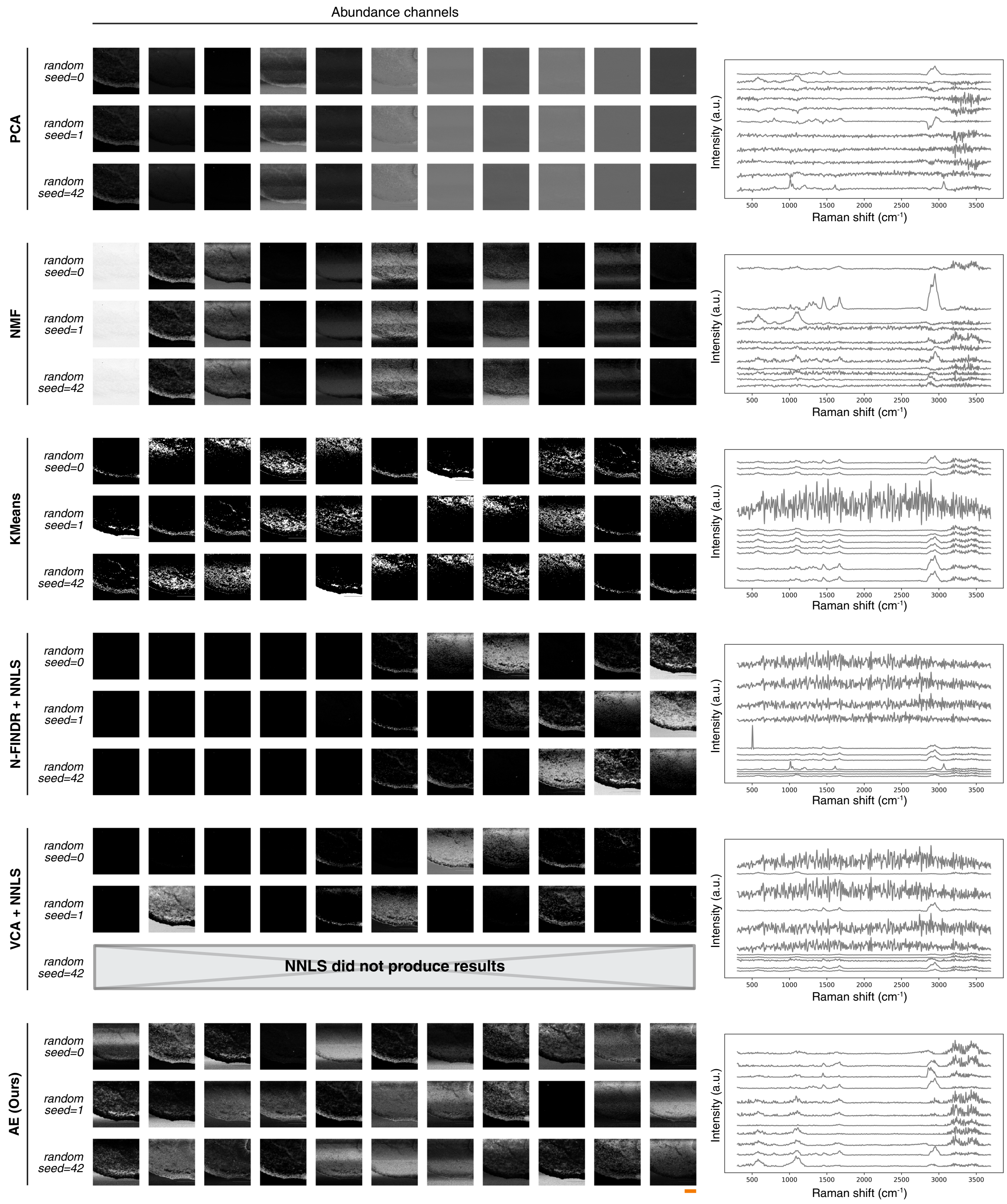

Figure S15: See next page for caption.

Figure S15: Comparative analysis of different chemometric methods on the Raman imaging scan of the second day-30 neural organoid cryosection in the presence of higher degrees of experimental noise (as shown in Figures 2B and 2D in the main text). Higher noise levels were simulated by applying a more conservative denoising procedure with a second-order Savitzky–Golay filter with a window size of 7 (98). Methods include: principal component analysis (PCA), non-negative matrix factorisation (NMF), k-means clustering, N-FINDR + non-negative least squares (NNLS), vertex component analysis (VCA) + NNLS, and our autoencoder (AE) pipeline. One of the analyses with VCA+NNLS failed to converge, with the NNLS implementation terminating after exceeding the maximum number of iterations. Abundance images were normalised for visualisation purposes. Endmember signatures displayed on the right correspond to the analyses performed with a random seed set to 42. Scale bar: 100 µm (bottom right, marked in orange). Raman data were acquired using the following parameters: pixel size = 2 µm in *x*-*y*, integration time = 0.5 second, laser power ≈ 48 mW, objective = 20×, NA 0.5. Created in BioRender. Georgiev, D. (2026). https://BioRender.com/3w9u235.

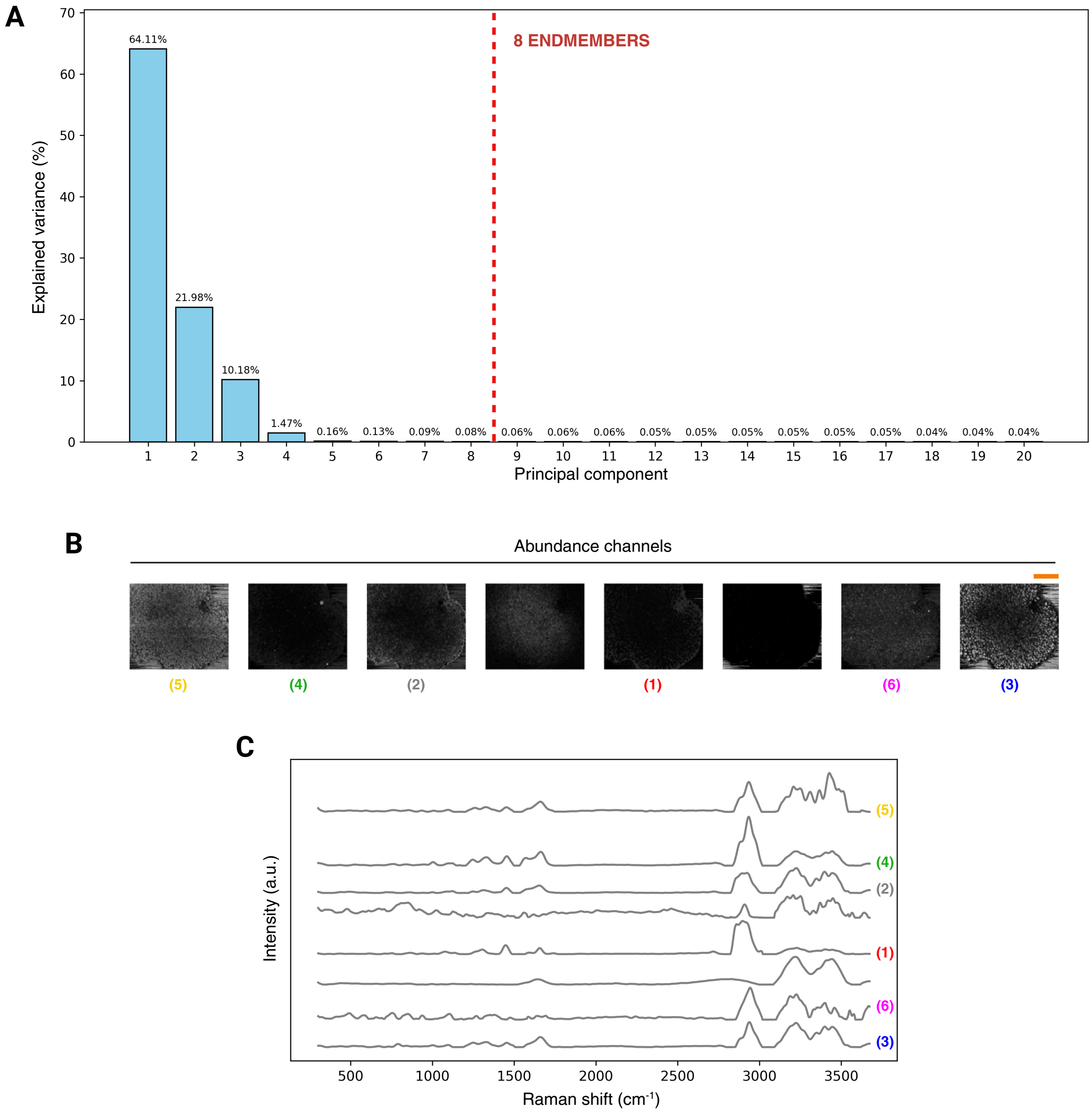

Figure S16: Extended unmixing results for the high-resolution Raman imaging scan of an intact day-10 neural organoid (as shown in Figure 3 in the main text). A, Principal component analysis of preprocessed RS data. The number of endmember components to extract was set to 8. **B,** Derived abundance images (normalised for visualisation purposes). Scale bar: 100 µm (top right, marked in orange). **C,** Corresponding endmembers. The endmember and abundance numbers indicate the components presented in the main text. Raman data were acquired using the following parameters: pixel size = 1 µm in *x*-*y*, integration time = 0.5 second, laser power ≈ 32 mW, objective = 63×, NA 1. Created in BioRender. Georgiev, D. (2026). https://BioRender.com/stsrquo.

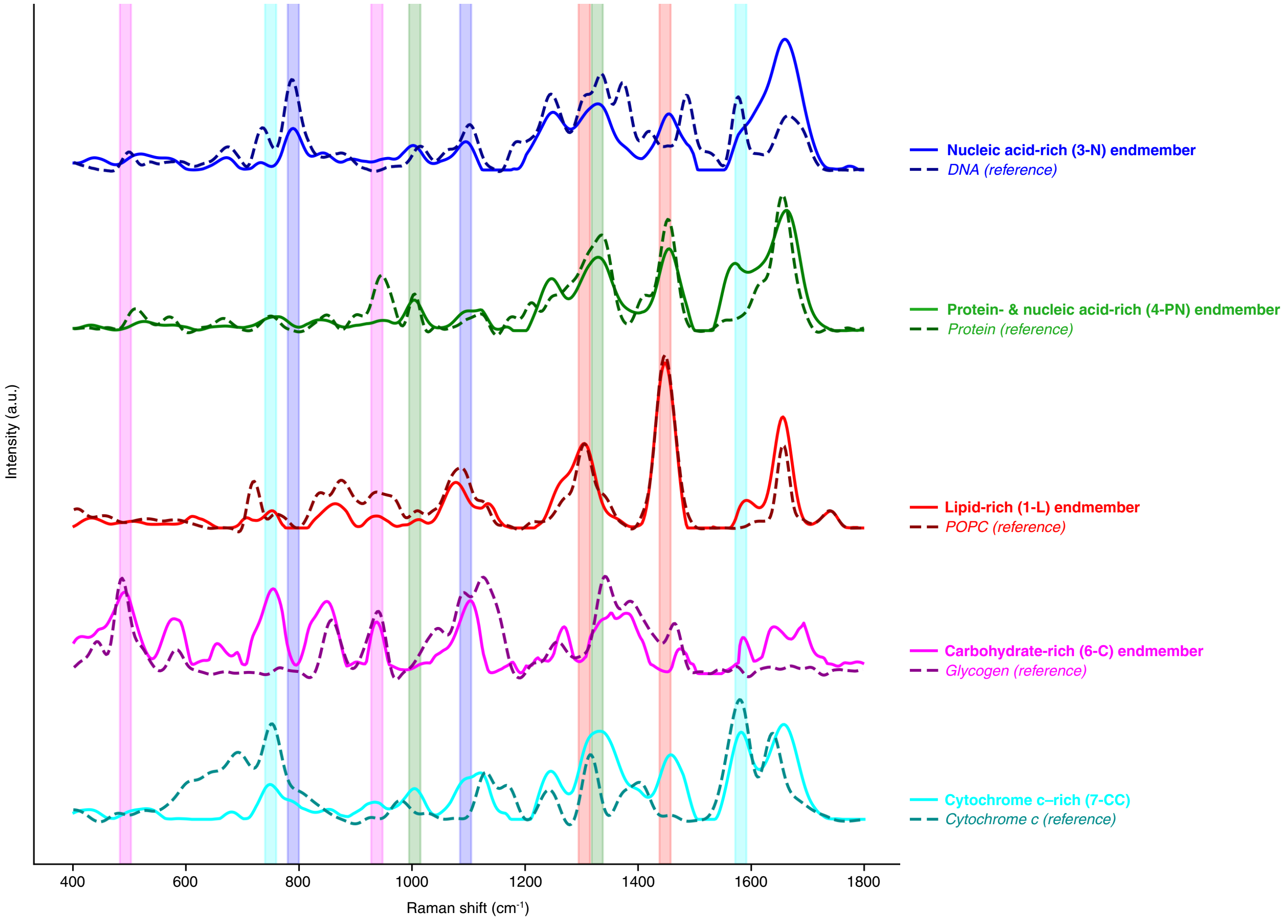

Figure S17: Characterisation of derived endmembers against reference spectra. Endmember spectra from Raman imaging in intact neural organoids – 3-N, 4-PN, 1-L and 6-C from Figure 3 and 7-CC from Figure 4, overlaid with reference spectra of DNA, protein, POPC, glycogen and cytochrome *c* from LaLone et al. (53). Shaded windows mark characteristic bands of each biomolecular class, shown in the same colour. Raman data were acquired using the following parameters: pixel size = 1 µm (3-N, 4-PN, 1-L and 6-C) or 2 µm (7-CC) in *x*-*y*, integration time = 0.5 second, laser power ≈ 32 mW, objective = 63×, NA 1. Created in BioRender. Georgiev, D. (2026). https://BioRender.com/1hv3k84.

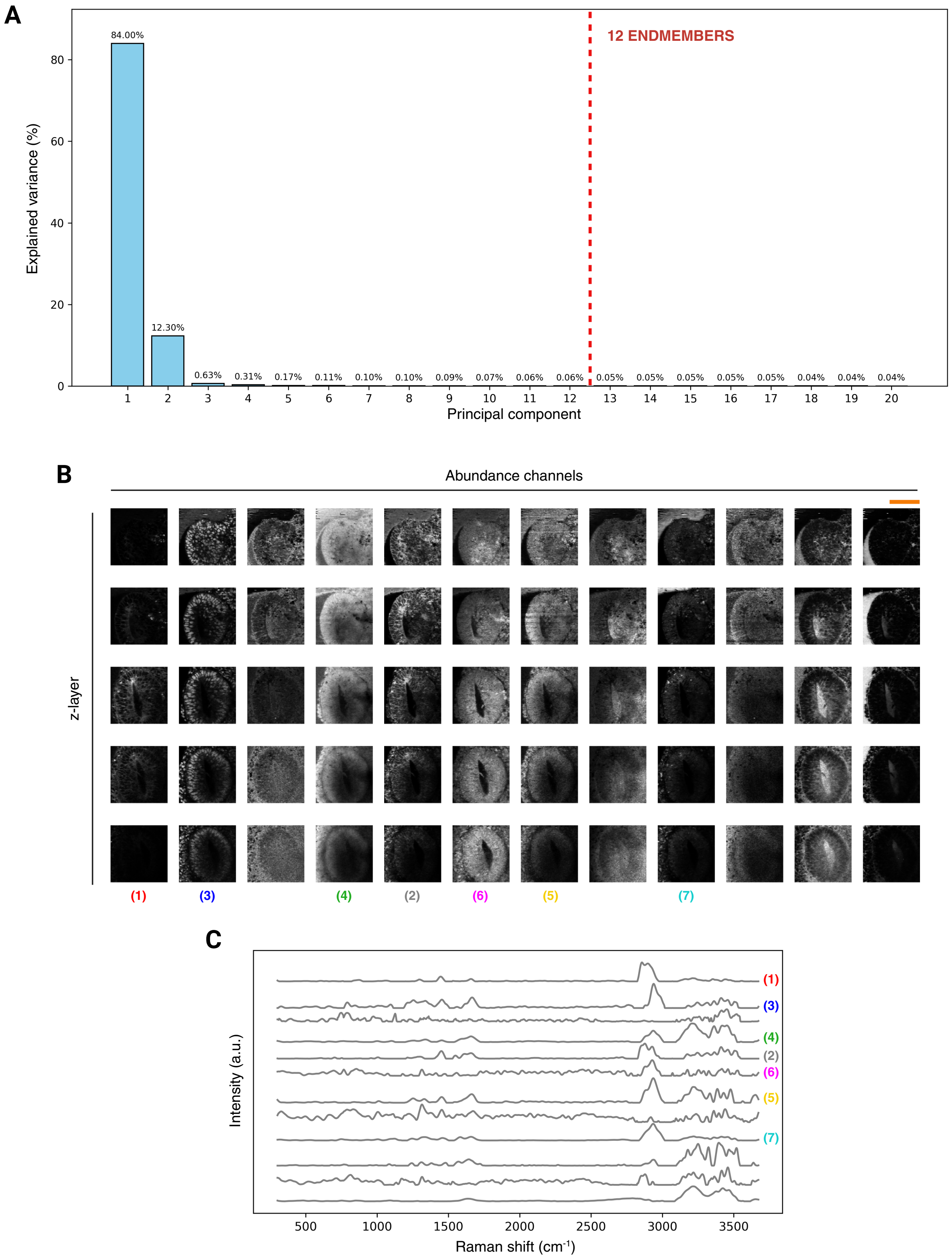

Figure S18: Extended unmixing results for the volumetric Raman imaging scan of a neural rosette within an intact day-20 neural organoid (as shown in Figures 4B–E in the main text). A, Principal component analysis of preprocessed RS data. The number of endmember components to extract was set to 12. **B,** Derived abundance images (normalised for visualisation purposes). Scale bar: 100 µm (top right, marked in orange). **C,** Corresponding endmembers. The endmember and abundance numbers indicate the components presented in the main text. Raman data were acquired using the following parameters: pixel size = 2 µm in *x*-*y* and 10 µm in *z*, integration time = 0.5 second, laser power ≈ 32 mW, objective = 63×, NA 1. Created in BioRender. Georgiev, D. (2026). https://BioRender.com/1qs8atn.

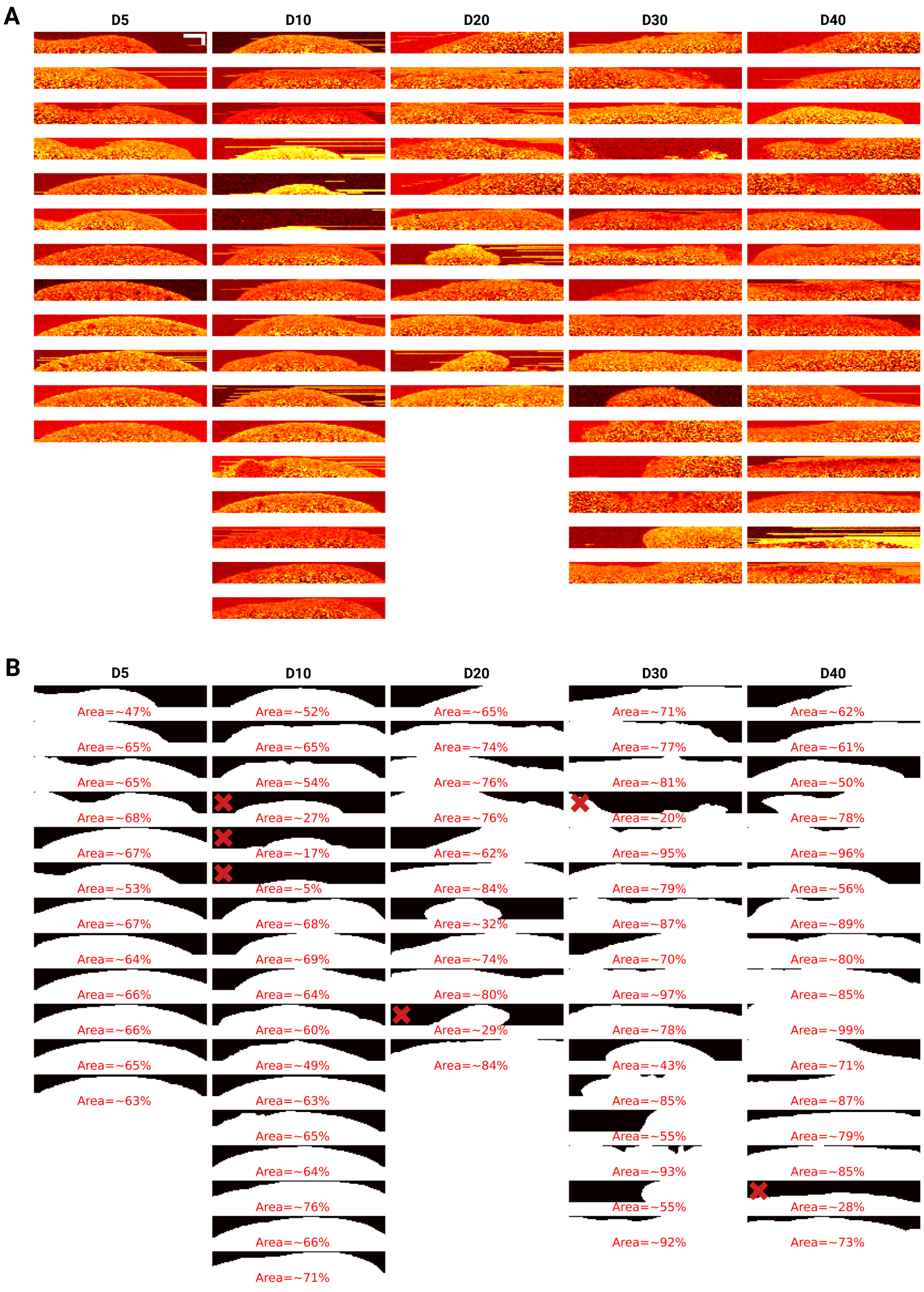

Figure S19: Preparation of timepoint Raman data for quantification analysis (as shown in Figures 5B–D in the main text). A, Intensity slices across the 1659 cm^{-1} band (linked to proteins and lipids) of the collected Raman imaging scans. Images were normalised for visualisation purposes. Scale bar: 100 µm (top left). **B,** Annotated organoid masks, along with estimated tissue coverage. Scans with tissue coverage below 30% were not included in quantification analysis (marked with a red cross). Raman data were acquired using the following parameters: pixel size = 5 µm in *x*-*y* and 10 µm in *z*, integration time = 0.5 second, laser power ≈ 32 mW, objective = 63×, NA 1. Created in BioRender. Georgiev, D. (2026). https://BioRender.com/ypzhc0q.

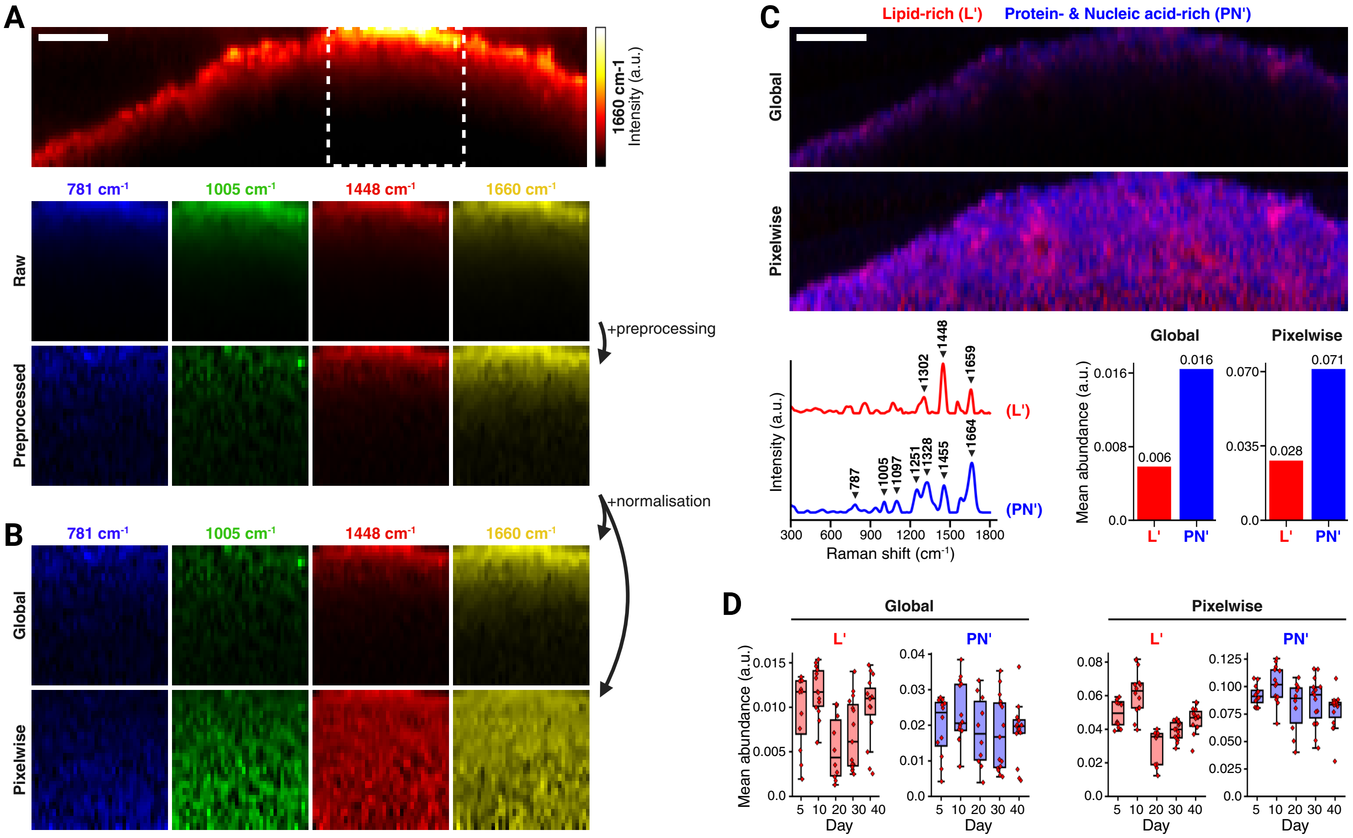

Figure S20: Effect of normalisation on unmixing performance – global versus pixelwise normalisation. A–B, Illustration of preprocessing and normalisation using a representative scan from our timepoint study (day-20 organoid presented in Figure 5D in the main text). **A,** Intensity profile across the 1660 cm^{-1} Raman shift (top) and corresponding images at four selected Raman shifts within a region of interest (white dashed box), shown before and after preprocessing (bottom). Shifts correspond to channels associated with nucleic acids (781 cm^{-1}), proteins (1005 cm^{-1} and 1660 cm^{-1}) and lipids (1448 cm^{-1} and 1660 cm^{-1}), selected based on derived endmembers (Figure 5B). **B,** Selected Raman bands after normalisation, comparing global (*top*) and pixelwise (*bottom*) approaches. **C–D,** Effect of normalisation on hyperspectral unmixing in timepoint data – global versus pixelwise normalisation. Unmixing was performed with our autoencoder pipeline, which was applied to both versions of the data combined together. **C,** Reconstruction of the selected scan following global versus pixelwise normalisation, alongside the corresponding endmember signatures and their mean abundance across the selected scan. **D,** Impact of global versus pixelwise normalisation on abundance quantification trends derived across the timepoint dataset. Raman data were acquired using the following parameters: pixel size = 5 µm in *x*-*y* and 10 µm in *z*, integration time = 0.5 second, laser power ≈ 32 mW, objective = 63×, NA 1. Created in BioRender. Georgiev, D. (2026). https://BioRender.com/tsxyic1.

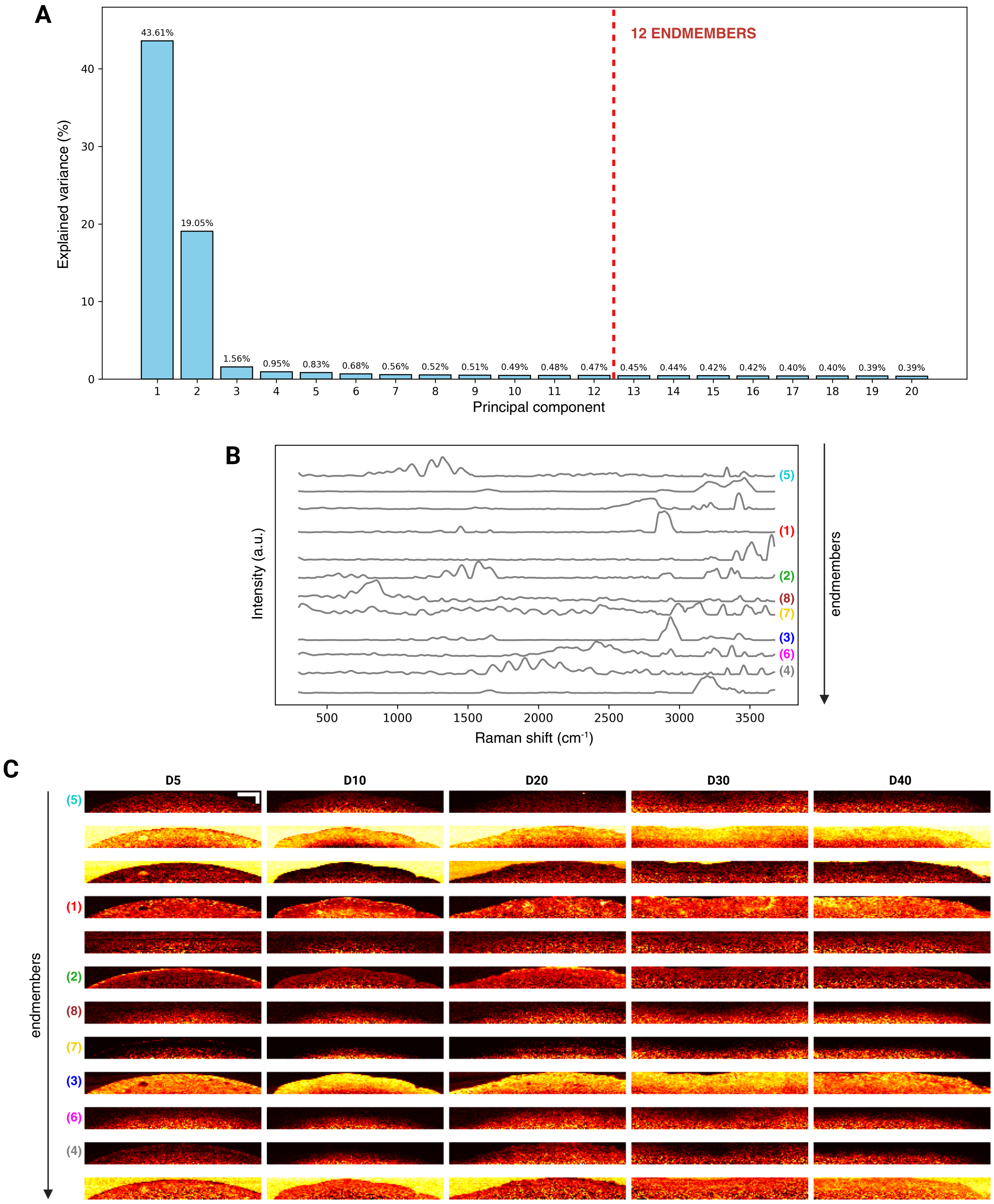

Figure S21: Extended unmixing results for the collected timepoint Raman imaging data (as shown in Figures 5B–D in the main text). A, Principal component analysis of preprocessed RS data. The number of endmember components to extract was set to 12. **B,** Derived endmembers. **C,** Corresponding abundance images (normalised for visualisation purposes) for five representative organoid samples. The endmember and abundance numbers indicate the components presented in the main text. Scale bar: 100 µm (top left). Abundance reconstructions of components marked in red (1) and blue (3) for all organoid scans are presented in Figures S23A and S23B, respectively. Raman data were acquired using the following parameters: pixel size = 5 µm in x-y and 10 µm in z, integration time = 0.5 second, laser power ≈ 32 mW, objective = 63×, NA 1. Created in BioRender. Georgiev, D. (2026). https://BioRender.com/8tow6io.

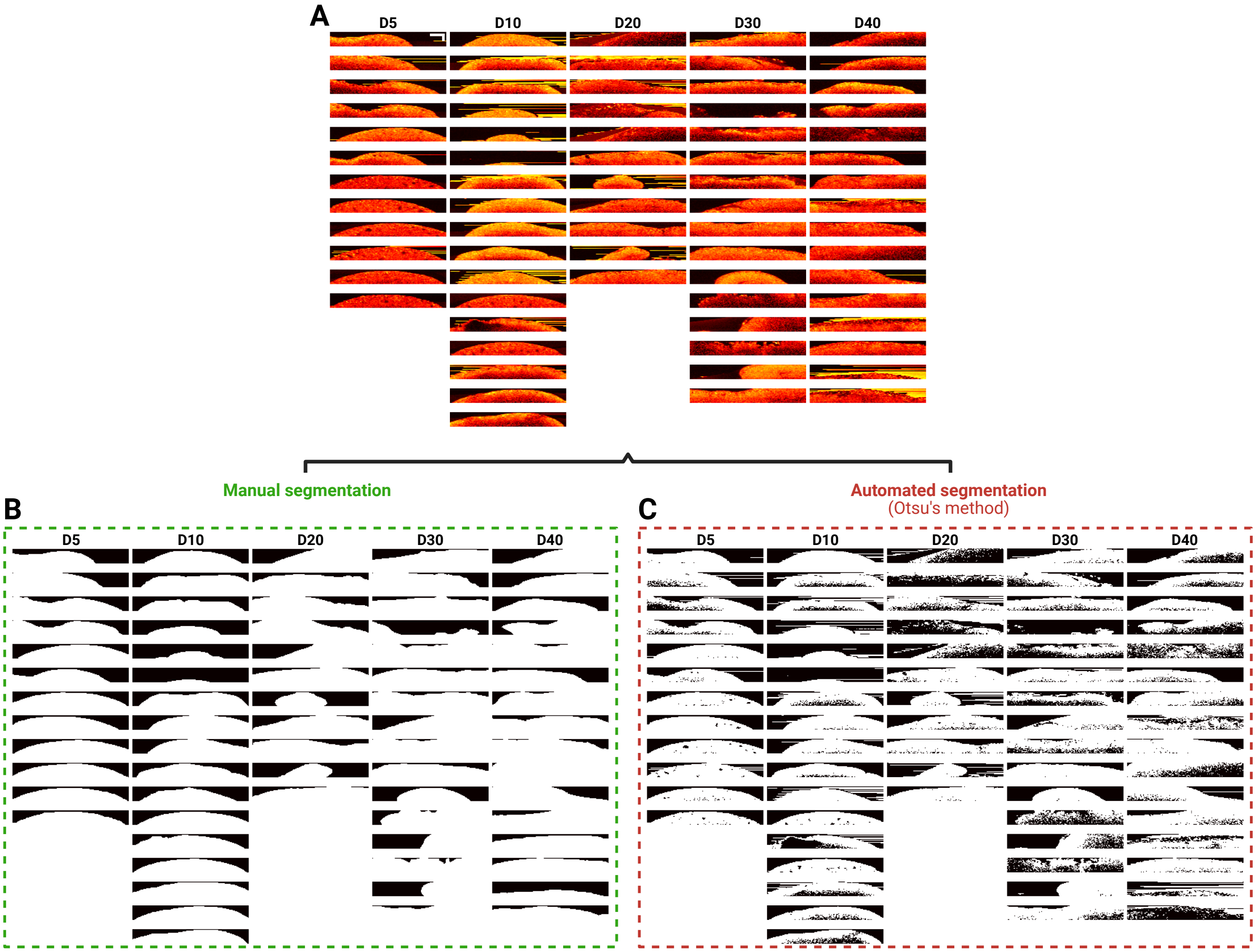

Figure S22: Manual versus automated tissue segmentation of timepoint scans (as shown in Figures 5B–D in the main text). A, Tissue-associated abundance maps used to guide segmentation (3-PN; protein- and nucleic acid-rich) of timepoint scans. Scale bar: 100 µm (top left). Data reused from Figure S23B. **B,** Corresponding manually delineated tissue masks used for quality control and abundance quantification. Data reused from Figure S19B. **C,** Automated masks obtained using Otsu thresholding on the abundance maps of 3-PN, illustrating frequent speckling, fragmentation and misclassified pixels. Raman data were acquired using the following parameters: pixel size = 5 µm in *x*-*y* and 10 µm in *z*, integration time = 0.5 second, laser power ≈ 32 mW, objective = 63×, NA 1. Created in BioRender. Georgiev, D. (2026). https://BioRender.com/ddz0vsx.

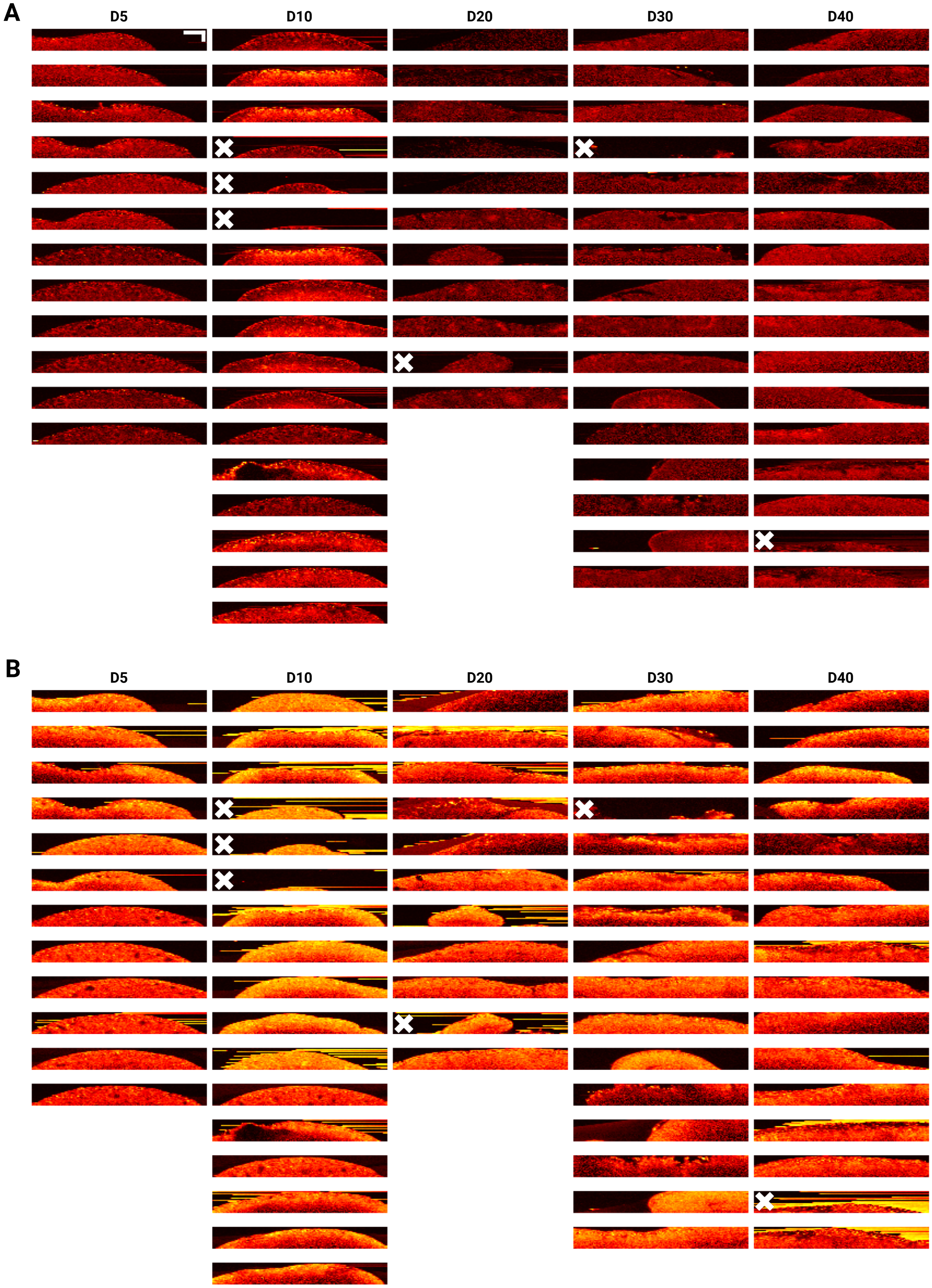

Figure S23: Abundance images of the two most abundant endmembers across timepoint RS scans (as shown in Figures 5B–D in the main text). A, Abundance images of endmember 1 (lipid-rich). Scale bar: 100 µm (top left). **B,** Abundance images of endmember 3 (protein- and nucleic acid-rich). Images were not normalised. Scans excluded from quantification analysis are marked with a white cross. Raman data were acquired using the following parameters: pixel size = 5 µm in x-y and 10 µm in z, integration time = 0.5 second, laser power ≈ 32 mW, objective = 63×, NA 1. Created in BioRender. Georgiev, D. (2026). https://BioRender.com/5cnhs83.

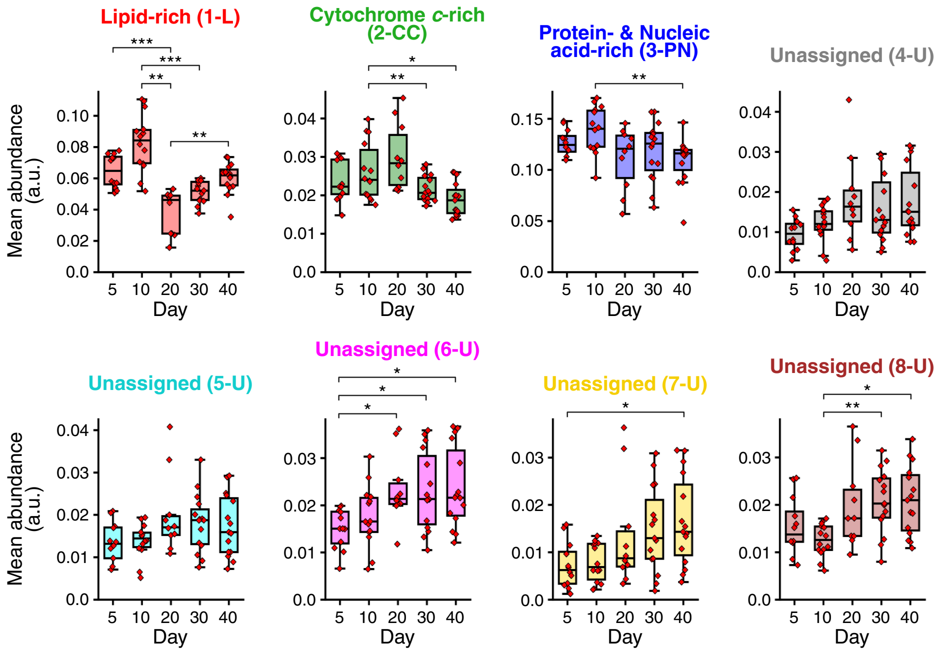

Figure S24: Statistical significance tests of abundance timepoint trajectories (as shown in Figure 5C in the main text). Quantification of the corresponding abundances over time. Each dot represents the mean abundance of a scan (1–3 scans per organoid sample). Sample sizes: day 5 ($n = 12$), day 10 ($n = 14$), day 20 ($n = 10$), day 30 ($n = 15$), day 40 ($n = 15$) from $N = 3$ independent organoid culture batches. Statistical testing was performed at the scan/ROI level using a Kruskal-Wallis test followed by post-hoc Dunn's pairwise comparisons with Holm correction applied within each endmember across all day-pair comparisons (* $p < 0.05$, ** $p < 0.01$, *** $p < 0.001$). Figure 5C shows the same boxplots but without statistical significance annotations. Raman data were acquired using the following parameters: pixel size = 5 µm in x-y and 10 µm in z, integration time = 0.5 second, laser power ≈ 32 mW, objective = 63×, NA 1. Created in BioRender. Georgiev, D. (2026). https://BioRender.com/gqv3264.

REFERENCES

1. J. D. Bancroft, M. Gamble, *Theory and Practice of Histological Techniques* (Elsevier Health Sciences, 2008).

2. P. W. Harms, T. L. Frankel, M. Moutafi, A. Rao, D. L. Rimm, J. M. Taube, D. Thomas, M. P. Chan, L. Pantanowitz, Multiplex immunohistochemistry and immunofluorescence: A practical update for pathologists. *Mod. Pathol.* **36**, 100197 (2023).

3. B. N. G. Giepmans, S. R. Adams, M. H. Ellisman, R. Y. Tsien, The fluorescent toolbox for assessing protein location and function. *Science* **312**, 217–224 (2006).

4. P. L. Ståhl, F. Salmén, S. Vickovic, A. Lundmark, J. F. Navarro, J. Magnusson, S. Giacomello, M. Asp, J. O. Westholm, M. Huss, A. Mollbrink, S. Linnarsson, S. Codeluppi, Å. Borg, F. Pontén, P. I. Costea, P. Sahlén, J. Mulder, O. Bergmann, J. Lundeberg, J. Frisén, Visualization and analysis of gene expression in tissue sections by spatial transcriptomics. *Science* **353**, 78–82 (2016).

5. A. Rao, D. Barkley, G. S. França, I. Yanai, Exploring tissue architecture using spatial transcriptomics. *Nature* **596**, 211–220 (2021).

6. Y. Hasin, M. Seldin, A. Lusis, Multi-omics approaches to disease. *Genome Biol.* **18**, 83 (2017).

7. M. A. Lancaster, M. Renner, C.-A. Martin, D. Wenzel, L. S. Bicknell, M. E. Hurles, T. Homfray, J. M. Penninger, A. P. Jackson, J. A. Knoblich, Cerebral organoids model human brain development and microcephaly. *Nature* **501**, 373–379 (2013).

8. M. A. Lancaster, J. A. Knoblich, Organogenesis in a dish: Modeling development and disease using organoid technologies. *Science* **345**, 1247125 (2014).

9. S. P. Pașca, The rise of three-dimensional human brain cultures. *Nature* **553**, 437–445 (2018).

10. I. Chiaradia, M. A. Lancaster, Brain organoids for the study of human neurobiology at the interface of in vitro and in vivo. *Nat. Neurosci.* **23**, 1496–1508 (2020).

11. G. Quadrato, T. Nguyen, E. Z. Macosko, J. L. Sherwood, S. M. Yang, D. R. Berger, N. Maria, J. Scholvin, M. Goldman, J. P. Kinney, E. S. Boyden, J. W. Lichtman, Z. M. Williams, S. A. McCarroll, P. Arlotta, Cell diversity and network dynamics in photosensitive human brain organoids. *Nature* **545**, 48–53 (2017).

12. S. Velasco, A. J. Kedaigle, S. K. Simmons, A. Nash, M. Rocha, G. Quadrato, B. Paulsen, L. Nguyen, X. Adiconis, A. Regev, J. Z. Levin, P. Arlotta, Individual brain organoids reproducibly form cell diversity of the human cerebral cortex. *Nature* **570**, 523–527 (2019).

13. C. A. Trujillo, R. Gao, P. D. Negraes, G. Jing, J. Buchanan, S. Preissl, A. Wang, W. Wei, G. G. Haddad, I. A. Chaim, A. Domissy, M. Vandenberghe, A. Devor, G. W. Yeo, B. Voytek, A. R. Muotri, Complex oscillatory waves emerging from cortical organoids model early human brain network development. *Cell Stem Cell* **25**, 558–569.e7 (2019).

14. X. Qian, H. Song, G.-L Ming, Brain organoids: Advances, applications and challenges. *Development* **146**, dev166074 (2019).

15. E. Di Lullo, A. R. Kriegstein, The use of brain organoids to investigate neural development and disease. *Nat. Rev. Neurosci.* **18**, 573–584 (2017).

16. O. L. Eichmüller, J. A. Knoblich, Human cerebral organoids–A new tool for clinical neurology research. *Nat. Rev. Neurol.* **18**, 661–680 (2022).

17. Z. Yildirim, K. Swanson, W. Xuekun, J. Zou, W. Joseph, Next-gen therapeutics: Pioneering drug discovery with ipscs, genomics, ai, and clinical trials in a dish. *Annu. Rev. Pharmacol. Toxicol.* **65**, 71–90 (2024).

18. C. B. Martin, C. S. Chane, C. Clouchoux, A. Histace, Recent trends and perspectives in cerebral organoids imaging and analysis. *Front. Neurosci.* **15**, 629067 (2021).

19. R. Keshara, Y. H. Kim, A. Grapin-Botton, Organoid imaging: Seeing development and function. *Annu. Rev. Cell Dev. Biol.* **38**, 447–466 (2022).

20. K. Im, S. Mareninov, M. F. P. Diaz, W. H. Yong, *An Introduction to Performing Immunofluorescence Staining* (Springer, 2019).

21. U. Schnell, F. Dijk, K. A. Sjollema, B. N. G. Giepmans, Immunolabeling artifacts and the need for live-cell imaging. *Nat. Methods* **9**, 152–158 (2012).

22. L. Shi, M. Wei, Y. Miao, N. Qian, L. Shi, R. A. Singer, R. K. P. Benninger, W. Min, Highly-multiplexed volumetric mapping with Raman dye imaging and tissue clearing. *Nat. Biotechnol.* **40**, 364–373 (2022).

23. H. Tsurui, H. Nishimura, S. Hattori, S. Hirose, K. Okumura, T. Shirai, Seven-color fluorescence imaging of tissue samples based on Fourier spectroscopy and singular value decomposition. *J. Histochem. Cytochem.* **48**, 653–662 (2000).

24. F. Cutrale, V. Trivedi, L. A. Trinh, C.-L. Chiu, J. M. Choi, M. S. Artiga, S. E. Fraser, Hyperspectral phasor analysis enables multiplexed 5D in vivo imaging. *Nat. Methods* **14**, 149–152 (2017).

25. E. Fatti, S. Khawaja, K. Weis, The dark side of fluorescent protein tagging–The impact of protein tags on biomolecular condensation. *Mol. Biol. Cell* **36**, br10 (2025).

26. K. Dörner, M. J. Gut, D. Overwijn, F. Cao, M. Siketanc, S. Heinrich, N. Beuret, J. Meyer, T. Sharpe, K. Lindorff-Larsen, M. Hondele, Fluorescent protein and peptide tags alter condensate formation and dynamics in vivo and in vitro. *EMBO Rep.* **27**, 89–121 (2026).

27. N. Colthup, *Introduction to Infrared and Raman Spectroscopy* (Elsevier, 2012).

28. R. L. McCreery, *Raman Spectroscopy for Chemical Analysis* (John Wiley & Sons, 2005).

29. E. Smith, G. Dent, *Modern Raman Spectroscopy: A Practical Approach* (John Wiley & Sons, 2019).

30. H. J. Butler, L. Ashton, B. Bird, G. Cinque, K. Curtis, J. Dorney, K. Esmonde-White, N. J. Fullwood, B. Gardner, P. L. Martin-Hirsch, M. J. Walsh, M. R. McAinsh, N. Stone, F. L. Martin,

Using Raman spectroscopy to characterize biological materials. *Nat. Protoc.* **11**, 664–687 (2016).

31. K. Dodo, K. Fujita, M. Sodeoka, Raman spectroscopy for chemical biology research. *J. Am. Chem. Soc.* **144**, 19651–19667 (2022).

32. D. W. Shipp, F. Sinjab, I. Notingher, Raman spectroscopy: Techniques and applications in the life sciences. *Adv. Opt. Photonics* **9**, 315–428 (2017).

33. D. Cialla-May, X.-S. Zheng, K. Weber, J. Popp, Recent progress in surface-enhanced Raman spectroscopy for biological and biomedical applications: From cells to clinics. *Chem. Soc. Rev.* **46**, 3945–3961 (2017).

34. K. Kong, C. Kendall, N. Stone, I. Notingher, Raman spectroscopy for medical diagnostics–From in-vitro biofluid assays to in-vivo cancer detection. *Adv. Drug Deliv. Rev.* **89**, 121–134 (2015).

35. K. J. I. Ember, M. A. Hoeve, S. L. McAughtrie, M. S. Bergholt, B. J. Dwyer, M. M. Stevens, K. Faulds, S. J. Forbes, C. J. Campbell, Raman spectroscopy and regenerative medicine: A review. *NPJ Regen. Med.* **2**, 12 (2017).

36. I. Pence, A. Mahadevan-Jansen, Clinical instrumentation and applications of Raman spectroscopy. *Chem. Soc. Rev.* **45**, 1958–1979 (2016).

37. Á. Fernández-Galiana, O. Bibikova, S. V. Pedersen, M. M. Stevens, Fundamentals and applications of raman-based techniques for the design and development of active biomedical materials. *Adv. Mater.* **36**, 2210807 (2024).

38. A. Paudel, D. Raijada, J. Rantanen, Raman spectroscopy in pharmaceutical product design. *Adv. Drug Deliv. Rev.* **89**, 3–20 (2015).

39. L. R. Terry, S. Sanders, R. H. Potoff, J. W. Kruel, M. Jain, H. Guo, Applications of surface-enhanced Raman spectroscopy in environmental detection. *Anal. Sci. Adv.* **3**, 113–145 (2022).

40. S. R. Khandasammy, M. A. Fikiet, E. Mistek, Y. Ahmed, L. Halámková, J. Bueno, I. K. Lednev, Bloodstains, paintings, and drugs: Raman spectroscopy applications in forensic science. *Foren. Chem.* **8**, 111–133 (2018).

41. Z. Movasaghi, S. Rehman, I. U. Rehman, Raman spectroscopy of biological tissues. *Appl. Spectr. Rev.* **42**, 493–541 (2007).

42. A. C. S. Talari, Z. Movasaghi, S. Rehman, I. U. Rehman, Raman spectroscopy of biological tissues. *Appl. Spectr. Rev.* **50**, 46–111 (2015).

43. C. Krafft, B. Dietzek, J. Popp, Raman and CARS microspectroscopy of cells and tissues. *Analyst* **134**, 1046–1057 (2009).

44. T. Huser, J. Chan, Raman spectroscopy for physiological investigations of tissues and cells. *Adv. Drug Deliv. Rev.* **89**, 57–70 (2015).

45. G. Pezzotti, Raman spectroscopy in cell biology and microbiology. *J. Raman Spectr.* **52**, 2348–2443 (2021).

46. R. Smith, K. L. Wright, L. Ashton, Raman spectroscopy: An evolving technique for live cell studies. *Analyst* **141**, 3590–3600 (2016).

47. C.-C. Hsu, J. Xu, B. Brinkhof, H. Wang, Z. Cui, W. E. Huang, H. Ye, A single-cell Raman-based platform to identify developmental stages of human pluripotent stem cell-derived neurons. *Proc. Natl. Acad. Sci. U.S.A.* **117**, 18412–18423 (2020).

48. A. Ghita, F. C. Pascut, M. Mather, V. Sottile, I. Notingher, Cytoplasmic RNA in undifferentiated neural stem cells: A potential label-free Raman spectral marker for assessing the undifferentiated status. *Anal. Chem.* **84**, 3155–3162 (2012).

49. A. Ghita, F. C. Pascut, V. Sottile, C. Denning, I. Notingher, Applications of Raman micro-spectroscopy to stem cell technology: Label-free molecular discrimination and monitoring cell differentiation. *EPJ Tech. Instrum.* **2**, 6 (2015).

50. J. Geng, W. Zhang, C. Chen, H. Zhang, A. Zhou, Y. Huang, Tracking the differentiation status of human neural stem cells through label-free Raman spectroscopy and machine learning-based analysis. *Anal. Chem.* **93**, 10453–10461 (2021).

51. A. Germond, Y. Panina, M. Shiga, H. Niioka, T. M. Watanabe, Following embryonic stem cells, their differentiated progeny, and cell-state changes during iPS reprogramming by Raman spectroscopy. *Anal. Chem.* **92**, 14915–14923 (2020).

52. G. Bruno, M. Lipinski, K. J. Kobayashi-Kirschvink, C. Tentellino, P. T. C. So, J. W. Kang, F. De Angelis, Label-free detection of biochemical changes during cortical organoid maturation via Raman spectroscopy and machine learning. *Anal. Chem.* **97**, 5029–5037 (2025).

53. V. LaLone, A. Aizenshtadt, J. Goertz, F. S. Skottvoll, M. B. Mota, J. You, X. Zhao, H. E. Berg, J. Stokowiec, Y. Minzhi, A. Schwendeman, H. Scholz, S. R. Wilson, S. Krauss, M. M. Stevens, Quantitative chemometric phenotyping of three-dimensional liver organoids by Raman spectral imaging. *Cell Rep. Methods* **3**, 100440 (2023).

54. C. L. Lawson, R. J. Hanson, *Solving least squares problems* (SIAM, 1995).

55. D. Georgiev, Á. Fernández-Galiana, S. V. Pedersen, G. Papadopoulos, R. Xie, M. M. Stevens, M. Barahona, Hyperspectral unmixing for Raman spectroscopy via physics-constrained autoencoders. *Proc. Natl. Acad. Sci. U.S.A.* **121**, e2407439121 (2024).

56. D. Georgiev, S. V. Pedersen, R. Xie, A. Fernández-Galiana, M. M. Stevens, M. Barahona, RamanSPy: An open-source Python package for integrative Raman spectroscopy data analysis. *Anal. Chem.* **96**, 8492–8500 (2024).

57. N. Keshava, J. F. Mustard, Spectral unmixing. *IEEE Signal Process. Mag.* **19**, 44–57 (2002).

58. X. Li, R. Zhou, X. Yifei, X. Wei, Y. He, Spectral unmixing combined with Raman imaging, a preferable analytic technique for molecule visualization. *Appl. Spectr. Rev.* **52**, 417–438 (2017).

59. F. A. Kruse, A. B. Lefkoff, J. W. Boardman, K. B. Heidebrecht, A. T. Shapiro, P. J. Barloon, A. F. H. Goetz, The spectral image processing system (SIPS)–interactive visualization and analysis of imaging spectrometer data. *Remote Sens. Environ.* **44**, 145–163 (1993).

60. K. Pearson, Liii. On lines and planes of closest fit to systems of points in space. *Lond. Edinb. Dublin Philos. Mag. J. Sci.* **2**, 559–572 (1901).

61. H. Hotelling, Analysis of a complex of statistical variables into principal components. *J. Educ. Psychol.* **24**, 417–441 (1933).

62. D. D. Lee, H. Sebastian, Seung., Learning the parts of objects by non-negative matrix factorization. *Nature* **401**, 788–791 (1999).

63. S. Lloyd, Least squares quantization in PCM. *IEEE Trans. Inf. Theory* **28**, 129–137 (1982).

64. J. MacQueen, Some methods for classification and analysis of multivariate observations, in *Proceedings of the Fifth Berkeley Symposium on Mathematical Statistics and Probability, Volume 1: Statistics* (University of California Press, 1967), vol. 5, pp. 281–298.

65. M. E. Winter, N-FINDR: An algorithm for fast autonomous spectral end-member determination in hyperspectral data, in *Imaging Spectrometry V* (SPIE, 1999), vol. 3753, pp. 266–275.

66. J. M. P. Nascimento, J. M. B. Dias, Vertex component analysis: A fast algorithm to unmix hyperspectral data. *IEEE Trans. Geosci. Remote Sens.* **43**, 898–910 (2005).

67. Z. Wang, A. C. Bovik, H. R. Sheikh, E. P. Simoncelli, Image quality assessment: From error visibility to structural similarity. *IEEE Trans. Image Process.* **13**, 600–612 (2004).

68. T. Eissa, L. Voronina, M. Huber, F. Fleischmann, M. Žigman, The perils of molecular interpretations from vibrational spectra of complex samples. *Angew. Chem. Int. Ed. Engl.* **63**, e202411596 (2024).

69. M. Miotto, M. Rosito, M. Paoluzzi, V. de Turris, V. Folli, M. Leonetti, G. Ruocco, A. Rosa, G. Gosti, Collective behavior and self-organization in neural rosette morphogenesis. *Front. Cell Develop. Biol.* **11**, 1134091 (2023).

70. W. Masselink, D. Reumann, P. Murawala, P. Pasierbek, Y. Taniguchi, F. Bonnay, K. Meixner, J. A. Knoblich, E. M. Tanaka, Broad applicability of a streamlined ethyl cinnamate-based clearing procedure. *Development* **146**, dev166884 (2019).

71. A. A. Pollen, A. Bhaduri, M. G. Andrews, T. J. Nowakowski, O. S. Meyerson, M. A. Mostajo-Radji, E. Di Lullo, B. Alvarado, M. Bedolli, M. L. Dougherty, I. T. Fiddes, Z. N. Kronenberg, J. Shuga, A. A. Leyrat, J. A. West, M. Bershteyn, C. B. Lowe, B. J. Pavlovic, S. R. Salama, D. Haussler, E. E. Eichler, A. R. Kriegstein, Establishing cerebral organoids as models of human-specific brain evolution. *Cell* **176**, 743–756.e17 (2019).

72. S. Fernandes, D. Klein, M. C. Marchetto, Unraveling human brain development and evolution using organoid models. *Front. Cell Develop. Biol.* **9**, 737429 (2021).

73. J. G. Camp, F. Badsha, M. Florio, S. Kanton, T. Gerber, M. Wilsch-Bräuninger, E. Lewitus, A. Sykes, W. Hevers, M. Lancaster, J. A. Knoblich, R. Lachmann, S. Pääbo, W. B. Huttner, B. Treutlein, Human cerebral organoids recapitulate gene expression programs of fetal neocortex development. *Proc. Natl. Acad. Sci. U.S.A.* **112**, 15672–15677 (2015).

74. A. Jain, G. Gut, F. Sanchis-Calleja, R. Tschannen, Z. He, N. Luginbühl, F. Zenk, A. Chrisnandy, S. Streib, C. Harmel, R. Okamoto, M. Santel, M. Seimiya, R. Holtackers, J. K. Rohland, S. M. J. Jansen, M. P. Lutolf, J. Gray Camp, B. Treutlein, Morphodynamics of human early brain organoid development. *Nature* **644**, 1010–1019 (2025).

75. D. Reumann, M. Colombini, P. Möseneder, A. Piszczek, J. A. Knoblich, A cost-and time-efficient method for high-throughput cryoprocessing and tissue analysis using multiplexed tissue molds. *Cell Rep. Methods* **5**, 101023 (2025).

76. A. Uzquiano, A. J. Kedaigle, M. Pigoni, B. Paulsen, X. Adiconis, K. Kim, T. Faits, S. Nagaraja, N. Antón-Bolaños, C. Gerhardinger, A. Tucewicz, E. Murray, X. Jin, J. Buenrostro, F. Chen, S.

Velasco, A. Regev, J. Z. Levin, P. Arlotta, Proper acquisition of cell class identity in organoids allows definition of fate specification programs of the human cerebral cortex. *Cell* **185**, 3770–3788.e27 (2022).

77. Á. Vértesy, O. L. Eichmüller, J. Naas, M. Novatchkova, C. Esk, M. Balmaña, S. Ladstaetter, C. Bock, A. von Haeseler, J. A. Knoblich, Gruffi: An algorithm for computational removal of stressed cells from brain organoid transcriptomic datasets. *EMBO J.* **41**, e111118 (2022).

78. M. Ramosaj, S. Madsen, V. Maillard, V. Scandella, D. Sudria-Lopez, N. Yuizumi, L. Telley, M. Knobloch, Lipid droplet availability affects neural stem/progenitor cell metabolism and proliferation. *Nat. Commun.* **12**, 7362 (2021).

79. G. Cermenati, N. Mitro, M. Audano, R. C. Melcangi, M. Crestani, E. De Fabiani, D. Caruso, Lipids in the nervous system: From biochemistry and molecular biology to patho-physiology. *Biochim. Biophys. Acta* **1851**, 51–60 (2015).

80. T. J. Tracey, S. E. Kirk, F. J. Steyn, S. T. Ngo, The role of lipids in the central nervous system and their pathological implications in amyotrophic lateral sclerosis, in *Seminars in Cell & Developmental Biology* (Elsevier, 2021), vol. 112, pp. 69–81.

81. C. Garrido, L. Galluzzi, M. Brunet, P. E. Puig, C. Didelot, G. Kroemer, Mechanisms of cytochrome c release from mitochondria. *Cell Death Diff.* **13**, 1423–1433 (2006).

82. M. Hüttemann, P. Pecina, M. Rainbolt, T. H. Sanderson, V. E. Kagan, L. Samavati, J. W. Doan, I. Lee, The multiple functions of cytochrome c and their regulation in life and death decisions of the mammalian cell: From respiration to apoptosis. *Mitochondrion* **11**, 369–381 (2011).

83. R. Coronel, E. Garcia-Moreno, E. Siendones, M. J. Barrero, B. Martinez-Delgado, C. Santos-Ocaña, I. Liste, M. V. Cascajo-Almenara, Brain organoid as a model to study the role of mitochondria in neurodevelopmental disorders: Achievements and weaknesses. *Front. Cell. Neurosci.* **18**, 1403734 (2024).

84. D. Polli, V. Kumar, C. M. Valensise, M. Marangoni, G. Cerullo, Broadband coherent Raman scattering microscopy. *Laser Photonics Rev.* **12**, 1800020 (2018).

85. F. Vernuccio, R. Vanna, C. Ceconello, A. Bresci, F. Manetti, S. Sorrentino, S. Ghislanzoni, F. Lambertucci, O. Motiño, I. Martins, G. Kroemer, I. Bongarzone, G. Cerullo, D. Polli, Full-spectrum CARS microscopy of cells and tissues with ultrashort white-light continuum pulses. *J. Phys. Chem. B* **127**, 4733–4745 (2023).

86. D. A. Whitaker, K. Hayes, A simple algorithm for despiking Raman spectra. *Chemom. Intel. Lab. Syst.* **179**, 82–84 (2018).

87. P. H. C. Eilers, A perfect smoother. *Anal. Chem.* **75**, 3631–3636 (2003).

88. P. H. C. Eilers, H. F. M. Boelens, Baseline correction with asymmetric least squares smoothing. *Leiden Univ. Med. Centre Rep.* **1**, 5 (2005).

89. I. Goodfellow, Y. Bengio, A. Courville, Autoencoders, in *Deep learning* (MIT Press, 2016), chap. 14, pp. 499–523.

90. K. He, X. Zhang, S. Ren, J. Sun, Delving deep into rectifiers: Surpassing human-level performance on imagenet classification, in *Proceedings of the IEEE International Conference on Computer Vision* (2015), pp. 1026–1034.

91. S. Ioffe, C. Szegedy, Batch Normalization: Accelerating Deep Network Training by Reducing Internal Covariate Shift, in *Proceedings of the 32nd International Conference on Machine Learning*, vol. 37 of *Proceedings of Machine Learning Research* (PMLR, 2015), pp. 448–456.

92. N. Srivastava, G. Hinton, A. Krizhevsky, I. Sutskever, R. Salakhutdinov, Dropout: A simple way to prevent neural networks from overfitting. *J. Mach. Learn. Res.* **15**, 1929–1958 (2014).

93. X. Glorot, Y. Bengio, Understanding the difficulty of training deep feedforward neural networks, in *Proceedings of the Thirteenth International Conference on Artificial Intelligence and Statistics* (JMLR Workshop and Conference Proceedings, 2010), pp. 249–256.

94. D. P. Kingma, J. Ba, Adam: A method for stochastic optimization, in *Proceedings of the 3rd International Conference on Learning Representations* (2015).

95. M. Abadi, P. Barham, J. Chen, Z. Chen, A. Davis, J. Dean, M. Devin, S. Ghemawat, G. Irving, M. Isard, M. Kudlur, J. Levenberg, R. Monga, S. Moore, D. G. Murray, B. Steiner, P. Tucker, V. Vasudevan, P. Warden, M. Wicke, Y. Yu, X. Zheng, TensorFlow: A system for large-scale machine learning, in *Proceedings of the 12th USENIX Conference on Operating Systems Design and Implementation* (OSDI'16, USA, USENIX Association, 2016) pp. 265–283

96. S. S. Khan, M. G. Madden, New similarity metrics for Raman spectroscopy. *Chemom. Intel. Lab. Syst.* **114**, 99–108 (2012).

97. A. Z. Samuel, R. Mukojima, S. Horii, M. Ando, S. Egashira, T. Nakashima, M. Iwatsuki, H. Takeyama, On selecting a suitable spectral matching method for automated analytical applications of Raman spectroscopy. *ACS Omega* **6**, 2060–2065 (2021).

98. A. Savitzky, M. J. E. Golay, Smoothing and differentiation of data by simplified least squares procedures. *Anal. Chem.* **36**, 1627–1639 (1964).
